# Regulatory Functions of Fatty Acid Synthase on Redox Homeostasis and Lipid-Immune Crosstalk in Primary Hepatocytes of *Cyprinus carpio var*. Jian: A Comprehensive Evaluation of Antioxidant Capacity, Fatty Acid Profiles and Transcriptomic Signaling

**DOI:** 10.3390/antiox15101237

**Published:** 2026-09-24

**Authors:** Gang Jiang, Yu Zhang, Qing Wang, Jiayi Sang, Wenrong Feng, Yuanfeng Xu, Jianlin Li, Yongkai Tang

**Affiliations:** 1 Key Laboratory of Freshwater Fisheries and Germplasm Resources Utilization, Ministry of Agriculture and Rural Affairs, Freshwater Fisheries Research Center, Chinese Academy of Fishery Sciences, Wuxi 214081, China; jianggang@ffrc.cn (G.J.);; 2 Wuxi Fisheries College, Nanjing Agricultural University, Wuxi 214081, China

**Keywords:** fatty acid synthase, oxidative stress, antioxidant enzymes, lipid metabolism, *Cyprinus carpio var.* Jian

## Abstract

Fatty acid synthase (FASN) is the rate-limiting core enzyme for de novo fatty acid biosynthesis in aquatic animals, yet its regulatory role in teleost cellular antioxidant defense and redox balance remains poorly characterized. In this study, we identified and bioinformatically characterized the complete coding sequence (CDS) of Jian carp *FASN*, which encodes a 2514-amino acid polypeptide containing five conserved polyketide synthase (PKS) catalytic domains and 188 phosphorylation sites. qRT-PCR tissue profiling revealed ubiquitous *FASN* transcription across all tested tissues, with the highest abundance in liver tissue. To decipher the physiological functions of *FASN*, siRNA-mediated functional depletion was performed in isolated primary Jian carp hepatocytes. Suppression of endogenous *FASN* expression induced markers of oxidative damage, evidenced by significantly reduced activities of superoxide dismutase (SOD), glutathione peroxidase (GPx), and catalase (CAT) (*p* < 0.05), alongside elevated malondialdehyde (MDA) levels (*p* < 0.05). GC-MS detected global declines in cellular saturated, monounsaturated and polyunsaturated fatty acids, especially C14:0, C16:0 and C18:0. Transcriptionally, the *6-PGD* was downregulated (*p* < 0.05), whereas the fatty acid oxidation gene *PPARα* was upregulated (*p* < 0.05). RNA-seq identified 13,546 differentially expressed genes, with enrichment analyses indicating transcriptional shifts associated with oxidative damage and NF-κB-related inflammatory signaling. A total of 23 hub genes related to lipid turnover and arachidonic acid metabolism were screened. Collectively, our results demonstrate that *FASN* acts as an essential upstream regulator of hepatic redox balance in Jian carp. *FASN* disrupts fatty acid pools and triggers metabolic–inflammatory reprogramming, offering new insights into lipid–antioxidant crosstalk in teleost hepatocytes.

## 1. Introduction

De novo fatty acid synthesis is an indispensable metabolic process governing energy storage, membrane composition, and cellular signaling across vertebrates. Fatty acid synthase (*FASN*) is the multifunctional catalytic enzyme responsible for the complete synthesis of short- and medium-chain saturated fatty acids from acetyl-CoA and malonyl-CoA precursors [1]. Mammalian research has firmly established that *FASN* activity is tightly coupled to cellular redox status. Excessive inhibition of FASN depletes cellular antioxidant capacity, accumulates reactive oxygen species (ROS), and exacerbates lipid peroxidation, ultimately inducing oxidative stress and inflammatory activation in mammalian hepatocytes and tumor cells [2,3,4].

In farmed teleosts, hepatic lipid metabolism directly determines flesh quality, growth performance, and disease resistance, and balanced antioxidant capacity is critical to mitigate oxidative damage induced by nutritional and environmental stressors [5,6,7]. Jian carp (*Cyprinus carpio var*. Jian) is the most economically vital freshwater aquaculture species in China, with annual production exceeding one million tons [8,9]; elucidating the molecular regulators of its hepatic redox balance carries high practical value for improving cultivation stress resistance. To date, existing teleost *FASN* studies (including golden pompano [5], mandarin fish [10] and Nile tilapia [11]) have merely described tissue expression patterns and simple lipid phenotypes, while mechanistic investigations linking *FASN* to cellular redox homeostasis and antioxidant defense are extremely limited. The teleost liver serves as the dual central organ for endogenous lipogenesis and phase I/II antioxidant detoxification, equipped with complete pentose phosphate pathway (PPP) machinery for both fatty acid synthesis and antioxidant recycling [12]. Our preliminary transcriptomic screening successfully obtained the complete coding sequence (CDS) of Jian carp *FASN*, laying a foundation for functional verification of its redox regulatory roles.

In the present study, we integrated bioinformatic CDS characterization, tissue expression profiling, siRNA-mediated gene knockdown in primary hepatocytes, fatty acid metabolomics, antioxidant biochemical detection, and RNA-seq transcriptomics to address three core redox-focused questions: (1) How does *FASN* deficiency alter the activity of key antioxidant enzymes and lipid peroxidation levels in carp hepatocytes? (2) What changes occur in cellular fatty acid pools upon disrupted *FASN* expression, and how do these lipids mediate oxidative damage? (3) What transcriptional signaling cascades connect *FASN* loss to oxidative stress and inflammatory immune activation? Our findings reveal a novel regulatory axis linking lipogenic *FASN* to teleost hepatic redox balance, offering new mechanistic insights into antioxidant regulation in freshwater fish.

## 2. Materials and Methods

### 2.1. Bioinformatics Analysis

The ORF Finder (https://www.ncbi.nlm.nih.gov/orffinder/) (accessed on 20 May 2026), BLASTX and BLASTN (http://www.ncbi.nlm.nih.gov/BLAST/) (accessed on 20 May 2026) from NCBI were used to infer amino acid sequences. BLAST searches were carried out on the non-redundant protein database with an *E*-value of 0.05. The online server TMHMM 2.0 (https://services.healthtech.dtu.dk/services/TMHMM-2.0/) (accessed on 20 May 2026) was adopted for the prediction of protein transmembrane regions. InterProScan (http://www.ebi.ac.uk/Tools/pfa/iprscan/) (accessed on 20 May 2026) was employed to identify conserved domains of the protein. The molecular weight and isoelectric point (pI) were calculated via the ProtParam tool (https://web.expasy.org/protparam/) (accessed on 20 May 2026). Signal peptide sequences and their cleavage sites were predicted using SignalP 5.0 Server (https://services.healthtech.dtu.dk/service.php?SignalP-5.0) (accessed on 20 May 2026) [13]. In addition, ProtScale (http://web.expasy.org/protscale/) (accessed on 20 May 2026) was applied to analyze the hydrophilicity and hydrophobicity profiles of the protein [14]. NetPhos 3.1 (https://services.healthtech.dtu.dk/services/NetPhos-3.1/) (accessed on 20 May 2026) was applied to predict phosphorylation sites of the protein. The secondary and tertiary structures were separately modeled via the Phyre2 Server (http://www.sbg.bio.ic.ac.uk/phyre2/) (accessed on 20 May 2026) and SWISS-MODEL [15]. Multiple sequence alignment among homologous sequences from different species was carried out using DNAMAN 8.0. Meanwhile, sequence alignment was also performed with ClustalW under default parameter settings [16]. Phylogenetic analysis was conducted in MEGA 4.0 using the neighbor-joining algorithm with 500 bootstrap replicates, and all gap-containing and incomplete sites were removed prior to tree construction [17].

### 2.2. Fish Preparation

The Jian carp used in the current study were procured from the Freshwater Fisheries Research Center (FFRC) farm, Wuxi city, China. The juveniles were maintained in a recirculating aquaculture system (RAS) and fed a commercial diet for 14 days to allow for acclimatization to the experimental conditions. During this period, Jian carp were fed the commercial diet twice a day (8: 30 and 16:00), with the amount of feed provided at 2% of their body weight.

### 2.3. Tissue Expression Analysis by Quantitative Real-Time PCR (qRT-PCR)

The mRNA expression levels of *FASN* were measured in various tissues of five Jian carp (13.74 ± 1.54 g, 7.84 ± 0.37 cm) by qPCR analysis. Fish were collected and anesthetized in eugenol solution (Aladdin, Shanghai, China) for 5 min before being sacrificed by dissection. The tissues tested, including brain, kidney, spleen, gill, liver, muscle and intestine, were sampled using the sharp tweezers and scissors, respectively. The samples were quickly placed into 1.5 mL centrifuge tubes, and immediately immersed in liquid nitrogen until use for RNA extraction.

Total RNA was extracted from specimens with TRIzol reagent (Sangon Biotech, Shanghai, China), and first-strand complementary DNA (cDNA) was reverse-transcribed utilizing the HiScript IV All-in-One Ultra RT SuperMix Kit (Vazyme, Nanjing, China). SYBR Green-based quantitative real-time PCR (qRT-PCR) amplification was performed on a CFX Opus 96 Real-Time PCR Detection System (Bio-Rad, Shanghai, China). All reaction protocols and subsequent data analytical pipelines complied with the official protocols supplied by the reagent manufacturers. All oligonucleotide primers applied for qRT-PCR quantification are summarized in Table 1. The β-actin transcript was chosen as the internal control gene, and relative gene expression levels were determined through the comparative 2^(−ΔΔCt)^ algorithm [18].

### 2.4. Primary Hepatocytes Isolation and Cultivation

Primary hepatocytes were isolated from the Jian carp as previously described by Le et al. (2017) [19] with minor modifications. Briefly, three healthy fish were anesthetized with eugenol (Aladdin, China), and then the body surface was wiped with 75% ethanol. Livers were aseptically excised and immediately placed in ice-cold phosphate-buffered saline (PBS) (Sangon Biotech, Shanghai, China) containing antibiotics (penicillin, 100 U/mL; streptomycin, 100 μg/mL). The tissue was rinsed once with 75% ethanol for 30 s, followed by three rinses with PBS. The liver was then minced into small pieces (1–3 mm^3^) using sterilized surgical scissors. Tissue pieces were placed into 25 cm ^2^ culture flasks (Corning, New York, NY, USA) with 1 mL of Dulbecco’s modified Eagle’s Medium (DMEM; Gibco, Miami, FL, USA) supplemented with 15% fetal bovine serum (FBS; Gibco, USA), 20 mM HEPES, 100 U/mL penicillin, and 100 μg/mL streptomycin, pH 7.4. Cultures were maintained at 26 °C in a standard atmosphere. After 24 h, an additional 3 mL of DMEM was added. Thereafter, half of the medium (2 mL) was replaced with fresh medium every two days. All siRNA transfection and downstream functional experiments were performed on non-passaged primary hepatocytes at 48 h after cell isolation. MitoTracker staining verified intact mitochondrial activity and good cellular condition of these primary hepatocytes.

### 2.5. siRNA Transfection

Small interfering RNA (siRNA) duplexes, specifically targeting distinct coding regions of the fatty acid synthase (*FASN*) gene, were designed and synthesized through chemical means by Sangon Biotech based in Shanghai, China. In this experiment, a non-targeting scrambled siRNA, referred to as siRNA-NC, served as a negative control to evaluate the efficacy of the targeted siRNA. The siRNA-NC represents scrambled non-targeting siRNA, which was transfected into hepatocytes following exactly the same transfection protocol as the FASN-targeting siRNA, serving as a negative control to rule out the off-target effects caused by siRNA transfection reagents. The sequences of the siRNA used in this study can be found in Table 1. For the transfection process, hepatocytes were cultured in six-well plates, with a seeding density of 1 × 10^6^ cells per well in Dulbecco’s Modified Eagle Medium (DMEM) supplemented with 10% fetal bovine serum (FBS). The cells were allowed to adhere for a duration of 12 h, achieving approximately 70% confluence at the time of transfection, which is considered optimal for effective uptake of the siRNA. To facilitate transfection, 100 pmol of the siRNA was combined with Lipofectamine™ 3000 (from Invitrogen, Carlsbad, CA, USA) in Opti-MEM medium, following the manufacturer’s specified guidelines. The resulting siRNA–lipid complex from Beyotime Biotechnology, Shanghai, China, was then added dropwise to the cells in a careful manner. Six hours after transfection, the culture medium was replaced with fresh complete medium to reduce potential cytotoxicity associated with the transfection reagents. Each experimental condition, including both the targeting siRNA and the siRNA-NC, was executed in triplicate to ensure reliable results. The efficiency of the transfection was routinely assessed by conducting a parallel transfection utilizing a fluorescently labeled control siRNA, specifically a Cy3-labeled variant, to monitor uptake and confirm successful transfection.

### 2.6. Mitochondrial Activity Assessment

To ensure cellular health and monitor potential non-specific cytotoxic effects of the transfection procedure, mitochondrial activity was assessed 48 h post-transfection. Cells were incubated with 100 nM MitoTracker Red CMXRos (Beyotime Biotechnology, China) in complete medium for 30 min at 28 °C in the dark. This cationic dye selectively accumulates in active mitochondria. After staining, cells were washed twice with pre-warmed PBS and immediately visualized using a fluorescence microscope (Nikon Eclipse Ti2, Tokyo, Japan) equipped with a TRITC filter cube (excitation/emission: 579/599 nm). Cells displaying a bright, punctate red fluorescence pattern throughout the cytoplasm were considered to have functionally active mitochondria.

### 2.7. Knockdown Efficiency Validation and Real-Time PCR

Cells were harvested 48 h post-transfection for downstream analyses. The procedures for total RNA extraction and cDNA synthesis were identical to those described in Section 2.3 above. The knockdown efficiency of each siRNA was quantified by qPCR analysis of *FASN* mRNA expression, normalized to a β-actin, using the 2^(−ΔΔCt)^ method. Furthermore, genes related to fatty acid synthesis in cells (*6-PGD* and *PPARa*,) were also analyzed from these two groups, and the sequences of qRT-PCR primers were provided in Table 1.

### 2.8. Fatty Acid Analysis

Cellular samples obtained from both the siRNA treatment group (*n* = 4) and the siRNA-NC group (*n* = 4) underwent two rinses with phosphate-buffered saline (PBS). Following this, the samples were gently shaken for 5 min and then centrifuged at 1200× *g* for an additional 5 min. Around 100 mg of the cell pellets were used for total fatty acid extraction in clean centrifuge tubes, utilizing a mixture of sulfuric acid and methanol (2 mL at a ratio of 2.5:1, *v*/*v*), adhering to the traditional extraction method established by Folch et al. (1957) [20]. Transesterified lipid derivatives were subsequently characterized by gas chromatography–mass spectrometry (GC-MS, Agilent, Santa Clara, CA, USA) fitted with an HP-5 capillary column (60 m × 0.25 mm i.d., film thickness = 0.25 μm), with minor adjustments made to a previously described analytical workflow [21]. A 2 μL aliquot of each sample was injected at a split ratio of 1:10, with helium carrier gas delivered at a constant flow rate of 0.8 mL/min. The initial oven temperature was held at 40 °C for 2 min, then ramped up to 240 °C at 8 °C/min and maintained at this final temperature for 15 min. The temperatures for the inlet and ion source were adjusted to 250 °C and 280 °C, respectively. Ionization via electron impact was conducted at 70 eV, and mass spectra were obtained over the mass range from 35 to 500 amu.

### 2.9. Transcriptome Sequencing and Analysis

Additional cell pellets harvested from the siRNA treatment group (n = 3) and siRNA-NC (*n* = 3) were submitted to Majorbio Bio-Pharm Technology Co., Ltd. (Shanghai, China) for RNA-seq transcriptome profiling. Total RNA was isolated from these specimens with TRIzol reagent (Invitrogen, USA). RNA purity was verified by measuring the OD_260_/OD_280_ absorbance ratio, where acceptable values ranged from 1.8 to 2.2. RNA integrity was further examined on a NanoDrop 2000 spectrophotometer (Thermo Fisher, Shanghai, China), with all retained samples required to exhibit an RQN value no less than 6.5. Only samples containing a minimum of 1 μg total RNA at a concentration ≥30 ng/μL were selected for subsequent cDNA library construction. Procedures for library preparation included the random fragmentation of RNA into segments of approximately 300 bp, the synthesis of double-stranded cDNA, polishing of terminal ends, and the ligation of sequencing adapters. The final libraries underwent paired-end sequencing of 150 bp on the Illumina HiSeq 2500 platform, conducted by Majorbio Bio-Pharm Technology Co., Ltd. based in Shanghai, China.

High-quality clean reads were mapped to the reference genome of Jian carp (accession: GCF_019924925.1) via the HiSat2 alignment algorithm [22], and gene expression abundances were quantified according to mapped read counts. The DESeq2 and DEGseq2 packages were adopted for differential expression screening, with differentially expressed genes (DEGs) filtered under the criteria of FDR < 0.05 and |log_2_FC| ≥ 1. Transcript expression abundances were normalized to transcripts per million (TPM) values to facilitate cross-sample comparison of DEG expression patterns.

Furthermore, Gene Ontology (GO) and Kyoto Encyclopedia of Genes and Genomes (KEGG) functional enrichment analyses were performed to detect GO terms and metabolic pathways significantly enriched among the DEGs. Genes from the entire transcriptome were used as the background population, and statistical significance was defined as a Bonferroni-adjusted *p*-value < 0.05. GO enrichment and KEGG pathway analyses were performed separately using Goatools and the SciPy module in Python (version 3.13).

### 2.10. Antioxidant Enzyme Activity and MDA Content Detection

After siRNA transfection, primary hepatocytes were harvested from the siRNA treatment and the siRNA-NC group, and lysed with cell lysis buffer for subsequent biochemical detection. Cell lysates were centrifuged at 12,000 rpm for 10 min at 4 °C to collect supernatants. A BCA protein assay kit (Cat. No. A045-4-2, Nanjing Jiancheng Bioengineering) was used to quantify the total protein concentration of each sample, and all antioxidant indicators were normalized to total protein content to eliminate differences in sample loading. All antioxidant enzyme activities and lipid peroxidation levels were detected using commercial assay kits (Nanjing Jiancheng Bioengineering Institute, Nanjing, China) following the manufacturer’s standard protocols, with three biological replicates set for each group.

### 2.11. Quantitative Real-Time PCR (qRT-PCR) Validation

Quantitative real-time PCR (qRT-PCR) was carried out on six randomly selected differentially expressed genes (DEGs) to verify the reliability of transcriptomic sequencing data. The identical RNA specimens subjected to Illumina RNA-seq were reused for this validation assay. First-strand cDNA was reverse-transcribed with a commercial cDNA synthesis kit (Cat. No. RR047A, Tokyo, Takara). Subsequently, 2 μL of diluted cDNA template was mixed with gene-specific primers and amplified via qPCR using a dedicated fluorescence quantification kit (Cat. No. RR820A, Takara). Sequences of primers corresponding to target genes and the internal reference β-actin are listed in Table 1. Relative mRNA expression abundances of all candidate DEGs were calculated in accordance with the analytical workflow outlined in Section 2.7.

### 2.12. Statistical Analysis

All experimental results were presented as mean ± standard error (SE). For comparisons between the two groups (siRNA-NC control versus *FASN*-silenced groups, including qPCR gene expression, antioxidant enzyme activities (SOD, GPx, CAT), MDA content, and cellular fatty-acid composition), an independent-samples Student’s *t*-test was used. One-way analysis of variance (ANOVA) followed by Duncan’s multiple range test was applied only to datasets with three or more experimental groups. Normality and homogeneity-of-variance assumptions were checked for all datasets prior to statistical analysis using SPSS version 27.0. A *p*-value < 0.05 was considered statistically significant for all tests.

## 3. Results

### 3.1. Bioinformatic and Phylogenetic Characterization of Jian Carp FASN Protein

The full-length transcript containing the complete 7545 bp *FASN* open reading frame (ORF) was retrieved from Jian carp transcriptomic data. The total transcript length reached 9175 bp, including 184 bp 5′-UTR, 1446 bp 3′-UTR and the 7545 bp ORF encoding a 2514-amino acid polypeptide (Figure S1).

According to ProtParam analysis, the putative molecular weight of the protein encoded by the *FASN* gene was 274,727.61 Da (≈274.7 kDa), and its isoelectric point was 6.07. A total of five domains were predicted by InterProScan, and the domains spanning amino acids 1–409, 495–789, 827–1012, 1545–1860 and 1892–2070 corresponded to the PKS-KS, PKS-AT, PKS-DH, PKS-ER, and PKS-KR domains, respectively (Figure S1). The amino acid sequence of FASN contains 188 phosphorylation sites (Figure 1); however, no signal peptide was found by SignalP 5.0 Server (Figure 2). Hydrophilicity analysis showed that FASN is a hydrophilic protein, and the peak (positive) and the negative regions represent the hydrophobic and hydrophilic regions, respectively (Figure 3). TMHMM revealed that *FASN* is not a transmembrane protein. The secondary structure of *FASN* was found to contain 27.0% α-helix, 17.0% β-strand, and 8.0%, random coil, respectively, with the remaining 48.0% comprising other structural elements such as turns and loops. Moreover, the tertiary structure prediction of FASN protein was consistent with that of the secondary structure prediction, which mainly consisted of α-helix and β-strand (Figure 4).

Blast searches showed that the similarity between the FASN protein sequence of Jian carp and *Cyprinus carpio* (ARO92273.1) was the highest (98.17%), followed by *Carassius auratus* (XP_026090570.1) (97.37%), *Carassius gibelio* (XP_052467694.1) (97.22%), *Carassius carassius* (XP_059387184.1) (97.18%), and *Sinocyclocheilus anshuiensis* (XP_016335695.1) (96.98%). The phylogenetic tree constructed on the basis of FASN amino acid sequence similarity (Figure 5) also showed that Jian carp first clustered with *C. carpio*, and then with *C. Carassius*, *C. auratus* and *C. gibelio* in one branch. The mammals are grouped into individual branch. These results revealed that the *FASN* protein is conserved in the evolutionary process of fish.

### 3.2. Tissue Expression Distribution of FASN

The expression levels of the *FASN* gene in the various tissues of Jian carp were examined by qRT-PCR. The results showed that the *FASN* mRNA was expressed in every tissue examined, including brain, kidney, spleen, gill, liver, muscle, intestine and heart (Figure 6). However, the *FASN* gene was mainly expressed in the liver and brain tissues. The liver had the highest expression, followed by the brain, and these levels were significantly different from those in other tested tissues (*p* < 0.05). No significant differences were observed among the kidney, spleen, gill, muscle, intestine and heart tissues (*p* > 0.05).

### 3.3. Altered Antioxidant Enzyme Activities and Elevated Lipid Peroxidation Following FASN Knockdown

MitoTracker fluorescence staining demonstrated detectable mitochondrial membrane potential in both the control and *FASN* knockdown groups (Figure 7A). This observation makes severe mitochondrial dysfunction originating from transfection toxicity less likely, yet subtle transfection-associated cytotoxicity cannot be fully excluded based solely on this qualitative staining. *FASN* mRNA abundance was significantly reduced in si-*FASN* hepatocytes (*p* < 0.01, Figure 7B), confirming successful gene suppression. Transcripts of the pentose phosphate pathway gene *6-PGD* were markedly downregulated (*p* < 0.01), while *PPARα* (the transcription factor driving fatty acid catabolism and compensatory stress response) was strongly upregulated (*p* < 0.01, Figure 7D). This reciprocal transcriptional reprogramming disrupts the cellular supply of reducing equivalents required for antioxidant enzyme function.

### 3.4. Changes in Antioxidant Enzyme Activities and Elevated Lipid Peroxidation upon FASN Silencing

Compared with the negative control group, silencing *FASN* significantly reduced the activities of three core antioxidant enzymes (Figure 8): SOD activity decreased significantly (*p* < 0.05), weakening the superoxide anion scavenging capacity; GPx activity was markedly inhibited (*p* < 0.05), impairing glutathione-dependent hydroperoxide elimination; CAT activity declined sharply (*p* < 0.05), reducing cellular hydrogen peroxide clearance efficiency. Concomitantly, MDA content—the direct marker of lipid peroxidation and oxidative damage—was significantly elevated in *FASN*-deficient hepatocytes (*p* < 0.05). These data suggest that endogenous *FASN* expression is essential for maintaining basal antioxidant capacity and preventing ROS-mediated lipid oxidative injury in carp hepatocytes.

### 3.5. FASN Depletion Causes Global Reduction in Cellular Fatty Acid Substrates

GC-MS fatty acid profiling revealed a universal decline in all fatty acid classes upon *FASN* suppression (Table 2). The primary *FASN* catalytic products C14:0, C16:0, and C18:0 exhibited highly significant reductions (*p* < 0.01). Total saturated fatty acids (∑SFA), monounsaturated fatty acids (∑MUFA), and polyunsaturated fatty acids (∑PUFA) all decreased significantly. Long-chain n-3 and n-6 polyunsaturated fatty acids (including DHA) were also depleted; PUFA depletion further exacerbates oxidative susceptibility, as polyunsaturated lipids are highly susceptible to ROS-initiated peroxidation cascades.

### 3.6. Transcriptome Sequencing Analysis

#### 3.6.1. Sequencing and Assembly

To elucidate the regulatory role of the *FASN* gene in lipid metabolism of Jian carp, transcriptome sequencing was performed on primary hepatocytes following siRNA-mediated *FASN* knockdown (siRNA group) alongside the siRNA-NC negative-control group, with three biological replicates per group. After filtering, transcriptome sequencing generated 240,618,302 bp clean reads, with Q 20 and Q 30 values higher than 99.11% and 95.1%, respectively (Table 3). The GC content ranged from 44.62% to 47.41%. These results suggested that the transcriptome data obtained from the carp hepatocytes were reliable.

#### 3.6.2. Analysis of DEGs in the Silence and Control Group

Principal component analysis (PCA) revealed a clear separation between the control and siRNA-treated groups along the PC1 axis, indicating substantial transcriptomic alterations induced by *FASN* silencing, with good clustering of replicates within each group (Figure 9A). Differential expression analysis identified a total of 13,546 significantly differentially expressed genes (DEGs) based on the criteria of |log_2_FC| ≥ 1 and FDR < 0.05, of which 4724 were upregulated and 8822 were downregulated in response to *FASN* knockdown (Figure 9B). Further analysis using a Venn diagram showed that 18,120 genes (73.42%) were co-expressed in both groups, while 1359 genes (5.51%) were uniquely expressed in the control group and 5201 genes (21.07%) were exclusively detected in the siRNA-treated group (Figure 9C). Collectively, these results demonstrate that *FASN* silencing profoundly reshapes the transcriptional landscape of carp hepatocytes.

#### 3.6.3. Functional Enrichment Analysis

To further explore the functional implications of the uniquely expressed genes identified in the control group (5201 genes) and the *FASN*-silenced group (1359 genes), Gene Ontology (GO) and KEGG pathway enrichment analyses were performed. GO analysis at Level 2 revealed that genes uniquely expressed in the control group were primarily enriched in metabolic processes, cellular processes, cell part, organelle, binding, and catalytic activities, reflecting the active metabolic state of normal hepatocytes (Figure 10A). In contrast, genes specifically expressed in the *FASN*-silenced group were predominantly enriched in oxidative stress response, membrane lipid remodeling, and inflammatory biological processes (Figure 10B). KEGG pathway analysis further demonstrated distinct enrichment patterns: control-specific genes were most significantly enriched in Signal transduction, Transport and catabolism, and Cellular community—eukaryotes, with Lipid metabolism being the primary metabolism-related pathway (Figure 10C); siRNA-specific genes were most significantly enriched in Signaling molecules and interaction, Signal transduction, and Cell growth and death, with Lipid metabolism also emerging as the main metabolism-related pathway (Figure 10D).

#### 3.6.4. Identification of Oxidative Stress-Related Pathways and Genes Regulated by FASN Gene Silencing

Transcriptome analysis revealed that *FASN* silencing in primary carp hepatocytes induced significant transcriptional reprogramming, marked by the downregulation of key lipogenic gene *ACACA*, gluconeogenic gene *PCK*, and glyoxylate/dicarboxylate metabolism gene *GRHPR*. Concurrently, alterations were observed in genes governing fatty acid (*LIPC*), glycerophospholipid (*LPCAT1*), sphingolipid (*ENPP6*, *GM2A*, *SGMS*), and arachidonic acid (*PTGES*, *PLA2G*, *ALOX12*) metabolism, as well as protein palmitoylation (*PPT*). Moreover, robust activation of immune-related pathways was evident, including chemokine signaling (*CCL25*, *CCR9*, *CCR7*, *CCL22*), cytokine–cytokine receptor interaction (*IL12A*, *IL1B*, *TNF*, *IL6*), and the NF-kappa B pathway (*TRAF2*), alongside perturbation of cell cycle (*SGOL1*) (Table 4). These findings suggest that *FASN* knockdown not only disrupts multiple facets of hepatic lipid homeostasis but also triggers a coordinated inflammatory response in Jian carp.

#### 3.6.5. mRNA Expression Profiling of Lipid- and Inflammation-Associated Candidate Genes

Based on the clustering analysis shown in Figure 11, the heatmap clearly segregates the control and *FASN*-silenced groups into two distinct clusters, with the three biological replicates of each condition displaying high intra-group consistency. In the knockdown samples (Si1–Si3), a sharp reduction in transcript abundance is observed for *ACACA*, *PCK*, and *GRHPR* relative to the controls (C1–C3). Conversely, the majority of the remaining genes, including those involved in inflammatory responses (*CCL22*, *CCR7*, *CCR9*, *IL12A*, *IL1B*, *TNF*, *IL6*), lipid-related pathways (*ELOVL7*, *LIPC*, *ENPP6*, *SGMS*, *GM2A*, *ALOX12*, *PLA2G*), as well as *TRAF2* and *SGOL1*, show a pronounced increase in expression following *FASN* interference. This reciprocal expression pattern between metabolic repressors and immune-related activators further supports a functional shift from lipid/glucose metabolism toward inflammatory signaling upon *FASN* knockdown in carp hepatocytes.

#### 3.6.6. qRT-PCR Validation

To validate the reliability of the expression of DEGs identified via RNA-seq, six genes were randomly selected for validation using RT-qPCR (Figure 12). The qPCR results exhibited consistent expression patterns with those obtained from RNA-seq analysis. This confirmed the accuracy and reliability of the RNA-seq experiments.

## 4. Discussion

### 4.1. Structural Conservation and Tissue Expression of FASN Lay a Molecular Basis for Its Hepatic Redox Regulatory Function

In the present study, we retrieved the complete coding sequence (CDS) of Jian carp *FASN* from transcriptomic resources and performed systematic bioinformatic characterization of its encoded protein, providing structural evidence supporting the intrinsic linkage to *FASN* catalytic function. The 9175 bp *FASN* transcript encodes a 2514-amino-acid hydrophilic polypeptide without transmembrane or secretory signal sequences, confirming that *FASN* functions as an intracellular cytoplasmic enzyme responsible for cytosolic de novo lipogenesis, consistent with the subcellular localization of mammalian *FASN*. NetPhos prediction identified a total of 188 serine, threonine and tyrosine phosphorylation sites distributed across the full-length sequence. Post-translational phosphorylation is well-documented to modulate the catalytic efficiency of vertebrate *FASN* in mammalian models [23]: altered phosphorylation status changes the substrate binding affinity of PKS domains, and could indirectly shape cellular antioxidant capacity [24]. This abundant phosphorylation modification profile suggests that Jian carp *FASN* activity can be dynamically tuned under oxidative or nutritional stress, serving as a reversible molecular switch balancing lipogenesis and redox homeostasis.

Phylogenetic analysis revealed that Jian carp *FASN* shares the highest sequence identity (98.17%) with common carp and clusters tightly with other cyprinid species, while forming independent clades with mammals, amphibians and birds [25]. This evolutionary conservation indicates that the function of *FASN* is maintained across bony fish lineages, implying universal relevance of the *FASN*–redox axis in teleost hepatic physiology [26]. Tissue expression profiling via qRT-PCR further reinforced the liver as the core functional organ of *FASN*: *FASN* mRNA was detected in all tested tissues, but exhibited significantly higher abundance in the liver, followed by the brain [27]. The teleost liver is a dual-functional hub coordinating both de novo fatty acid synthesis and phase I/II antioxidant detoxification [28]. Brain *FASN* expression may support myelin lipid synthesis for neuronal membranes [27], where balanced redox status is critical to prevent lipid peroxidation of polyunsaturated neuronal lipids [29]. By contrast, peripheral tissues including muscle, kidney and gill displayed negligible *FASN* transcription, implying that these tissues rely largely on circulating exogenous lipids rather than endogenous *FASN*-mediated synthesis [25]. Prior fish studies only described tissue expression patterns of *FASN* without linking transcriptional distribution to antioxidant regulation; our expression data fills this gap and provides tissue-specific evidence that hepatic *FASN* is the primary mediator of lipid-redox balance in Jian carp.

### 4.2. FASN Depletion Suppresses Core Antioxidant Enzyme Activity

The most definitive finding of this work, aligned with the core scope of antioxidants, is that *FASN* silencing resulted in altered antioxidant enzyme profiles and elevated lipid peroxidation markers in primary Jian carp hepatocytes. Our biochemical measurements demonstrated significant declines in three rate-limiting antioxidant enzymes (SOD, GPx, CAT) alongside a prominent elevation of MDA, the canonical biomarker of lipid peroxidation and oxidative damage. This phenotype can be fully explained by the reciprocal transcriptional changes of *6-PGD* and *PPARα* observed upon *FASN* silencing.

*6-PGD* is the rate-limiting enzyme of the oxidative branch of the pentose phosphate pathway [30]. in vertebrate hepatocytes. In our *FASN*-knockdown model, transcriptional repression of *6-PGD* was observed. Importantly, transcript down-regulation does not necessarily correspond to reduced protein abundance or reduced catalytic activity, because we have not performed a biochemical measurement of *6-PGD* enzyme activity in the present work. Even so, the reduced *6-PGD* transcript, together with decreased SOD, GPx and CAT enzyme activities and increased MDA content, raises the hypothesis that transcriptional perturbation of the pentose phosphate pathway may be associated with impaired hepatic antioxidant capacity following *FASN* silencing. Further biochemical work will be needed to confirm functional changes in the pentose phosphate pathway in carp hepatocytes under *FASN* deficiency.

Consistent with this metabolic feedback loop, we observed compensatory upregulation of *PPARα*, a master transcription factor governing fatty acid *β*-oxidation. In mammalian hepatocytes, *PPARα* activation accelerates lipid catabolism to compensate for blocked de novo lipogenesis [31], yet enhanced fatty acid oxidation concurrently elevates mitochondrial ROS generation [32], exacerbating oxidative burden when antioxidant systems are already compromised [33]. MitoTracker staining showed preserved mitochondrial membrane potential in both groups, which argues against severe mitochondrial damage induced by transfection. Nevertheless, we cannot completely rule out mild transfection-related cytotoxicity, as no quantitative cell-viability assay was performed in this study.

### 4.3. FASN Suppression Reshapes Cellular Fatty Acid Pools and Increases Lipid Peroxidation Susceptibility

GC-MS fatty acid profiling revealed global depletion of saturated (SFA), monounsaturated (MUFA) and polyunsaturated (PUFA) fatty acids following FASN silencing, with the greatest reduction detected in C14:0, C16:0 and C18:0—the primary saturated fatty acid products catalyzed by *FASN*. This result aligns with the well-established catalytic function of *FASN*, which exclusively synthesizes short-to-medium chain saturated acyl chains as the foundational precursors for all downstream long-chain and unsaturated lipid species via elongase and desaturase enzymes [34].

The universal reduction of all fatty acid classes creates two interconnected risks to cellular redox stability, both relevant to lipid peroxidation [35]. First, depleted SFA substrates limit the de novo synthesis of membrane phospholipids, altering the structural integrity of hepatocyte plasma and organelle membranes [36]. Membranes deficient in saturated acyl chains exhibit increased fluidity, facilitating the diffusion of reactive oxygen species (ROS) across lipid bilayers and amplifying oxidative damage to intracellular compartments [37]. Second, residual polyunsaturated fatty acids (n-3 and n-6 PUFAs including DHA and ARA) within *FASN*-deficient cells become more prone to ROS-initiated peroxidation [38,39]. PUFAs contain multiple double bonds that are highly susceptible to free radical attack, generating cytotoxic lipid hydroperoxides that further raise MDA concentrations [40], matching our biochemical measurement of elevated MDA content in the knockdown group.

Additionally, transcriptome enrichment identified significant upregulation of arachidonic acid (ARA) metabolic pathways upon *FASN* loss. ARA, a key n-6 PUFA, acts as the precursor for pro-inflammatory eicosanoids (prostaglandins, leukotrienes) synthesized under oxidative stress conditions [41]. These eicosanoids amplify inflammatory signaling cascades, creating a positive feedback loop between lipid peroxidation, ROS accumulation and innate immune activation—an immunometabolic crosstalk thoroughly characterized in mammalian oxidative stress models but rarely reported in teleost hepatocytes [42]. Collectively, our fatty acid and transcriptome results indicate that *FASN* participates in the maintenance of hepatocyte redox homeostasis largely through sustaining balanced cellular fatty-acid pools, which constrains spontaneous lipid peroxidation and the generation of pro-oxidant eicosanoids.

### 4.4. Transcriptomic Reprogramming Links FASN Deficiency to Oxidative Stress-Mediated Inflammatory Signaling

Whole-transcriptome RNA-seq captured profound transcriptional remodeling triggered by FASN suppression, with 13,546 total differentially expressed genes (DEGs) clearly segregated by PCA between control and silencing groups. GO and KEGG functional enrichment revealed a dramatic transcriptional shift away from basal lipogenic and anabolic metabolism toward oxidative stress response, membrane lipid remodeling, cytokine signaling and cell cycle pathways—consistent with the physiological phenotype of impaired antioxidant defense and inflammatory activation suggested by our biochemical measurements.

Key lipogenic genes such as *ACACA* (acetyl-CoA carboxylase α), the upstream rate-limiting enzyme of *FASN*-mediated lipogenesis [43], were significantly downregulated alongside *FASN* itself, forming a coordinated suppression of the entire de novo fatty acid synthesis module. Concurrently, genes governing gluconeogenesis and glyoxylate metabolism (*PCK*, *GRHPR*) were also repressed, indicating global attenuation of carbon reductive biosynthesis when *FASN*-dependent lipid production is blocked. In contrast, a large suite of genes associated with oxidative stress and inflammation was transcriptionally activated, including chemokine ligands/receptors (*CCL22*, *CCR7*, *CCR9*), pro-inflammatory cytokines (*TNF*, *IL1B*, *IL6*), and the core NF-κB signaling mediator *TRAF2*. The NF-κB pathway is the master transcriptional switch for oxidative stress-triggered innate immunity in mammals. In the current study, we only detected transcriptional upregulation of NF-κB-related signaling genes; NF-κB protein translocation and post-translational activation were not experimentally assayed. Based on mammalian literature, ROS accumulation and lipid peroxidation products promote IκB phosphorylation and NF-κB nuclear translocation, driving the transcription of multiple pro-inflammatory mediators [44,45]. Our heatmap clustering results visually confirmed this reciprocal expression pattern between lipogenic repressors and inflammatory activators, which was independently validated via qRT-PCR on six randomly selected DEGs, verifying the reliability of transcriptomic profiling.

We further screened 23 hub genes bridging *FASN* function, lipid turnover, arachidonic acid metabolism, sphingolipid signaling and inflammatory cascades. Sphingolipid metabolic genes (*ENPP6*, *SGMS*, *GM2A*) were also enriched in DEGs; sphingolipids are critical lipid second messengers that transduce oxidative stress signals to initiate apoptosis and inflammatory responses [46], representing another layer linking disrupted *FASN* lipid synthesis to redox imbalance [47]. Collectively, the transcriptomic landscape provides genome-wide molecular evidence that *FASN* loss initiates a multi-layered stress response combining lipid pool dysregulation, markers of oxidative damage and transcriptional activation of NF-κB-associated inflammatory pathways, pointing toward a potential lipid–redox–immune regulatory axis in teleost hepatocytes.

Several key limitations of this study require acknowledgment. First, *FASN* knockdown was only verified at the mRNA level; species-specific antibodies and standardized activity assays for Jian carp were unavailable to confirm reduced FASN protein and catalytic activity. Even so, consistent downstream changes in antioxidant markers, cellular fatty acids and transcriptomes support the functional suppression of *FASN*. Second, our conclusions on oxidative stress and NF-κB-mediated inflammation rely solely on biochemical indicators and transcriptomics, without direct measurements of ROS, GSH redox state, or NF-κB/inflammatory protein levels. Third, mitochondrial health was evaluated only via qualitative MitoTracker staining, lacking quantitative viability assays; mild transfection-related cytotoxicity cannot be fully ruled out despite no obvious severe mitochondrial damage. Fourth, GC-MS fatty acid data were normalized to cell pellet wet weight rather than cell count or total protein, leaving minor cell mass differences unaccounted for. Nonetheless, dramatic declines in *FASN*-direct products (C14:0, C16:0, C18:0) confirm impaired de novo lipogenesis rather than non-specific biomass artifacts. Fifth, only one siRNA duplex was applied for *FASN* silencing with a single scrambled negative control; we did not test multiple independent siRNAs or rescue overexpression experiments to exclude off-target transcriptional effects.

Additional experimental constraints exist. All assays were conducted on isolated primary hepatocytes, which fail to mimic the intact in vivo hepatic microenvironment and systemic signals of Jian carp. Sample replicates for cell and omics tests were limited, and our findings remain unvalidated in live fish. Future work will validate *FASN* suppression at the protein/enzymatic level, quantify cell viability and redox/inflammatory protein signals, and replicate these phenotypes using in vivo carp models.

## 5. Conclusions

In summary, this study systematically clarifies the redox regulatory functions of *FASN* in primary Jian carp hepatocytes via CDS characterization, tissue expression screening, siRNA knockdown, antioxidant biochemical assays, fatty acid profiling and transcriptomic sequencing. Loss of *FASN* function triggers three interconnected oxidative disorders: (1) repression of pentose phosphate pathway-related gene 6-PGD occurs alongside reduced antioxidant-enzyme activities and elevated lipid-peroxidation markers, indicative of oxidative damage; (2) global depletion of saturated and polyunsaturated fatty acids disrupts membrane lipid homeostasis and increases cellular vulnerability to ROS damage; (3) and genome-wide transcriptional reprogramming triggers metabolic remodeling together with up-regulation of gene sets associated with NF-κB—linked inflammatory signaling. This work identifies *FASN* as a vital upstream modulator of teleost hepatic redox balance and suggests the existence of a novel lipid–antioxidant–immune crosstalk axis in cyprinid fish. Our results provide theoretical support for developing nutritional strategies to improve intrinsic antioxidant capacity and stress tolerance in freshwater farmed carp.

## Figures and Tables

**Figure 1:**
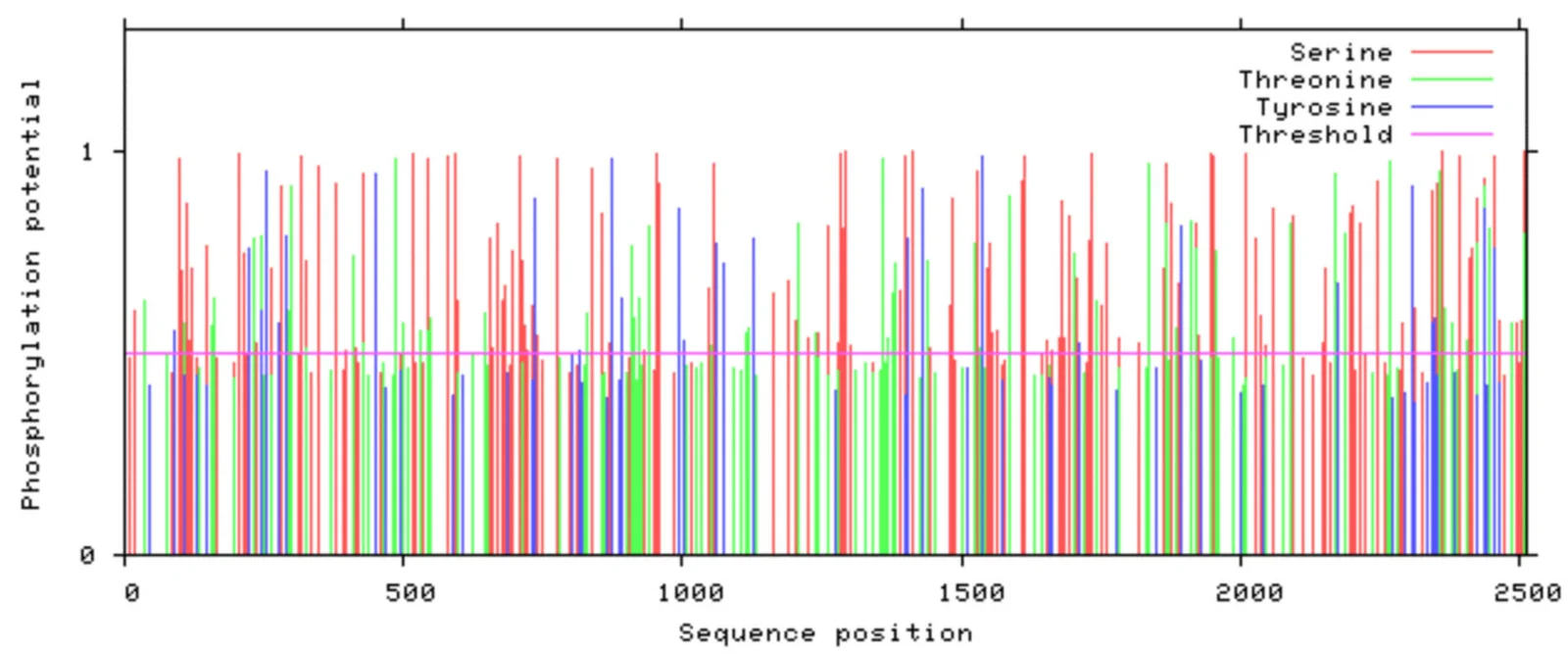
Phosphorylation site prediction of *FASN* protein.

**Figure 2:**
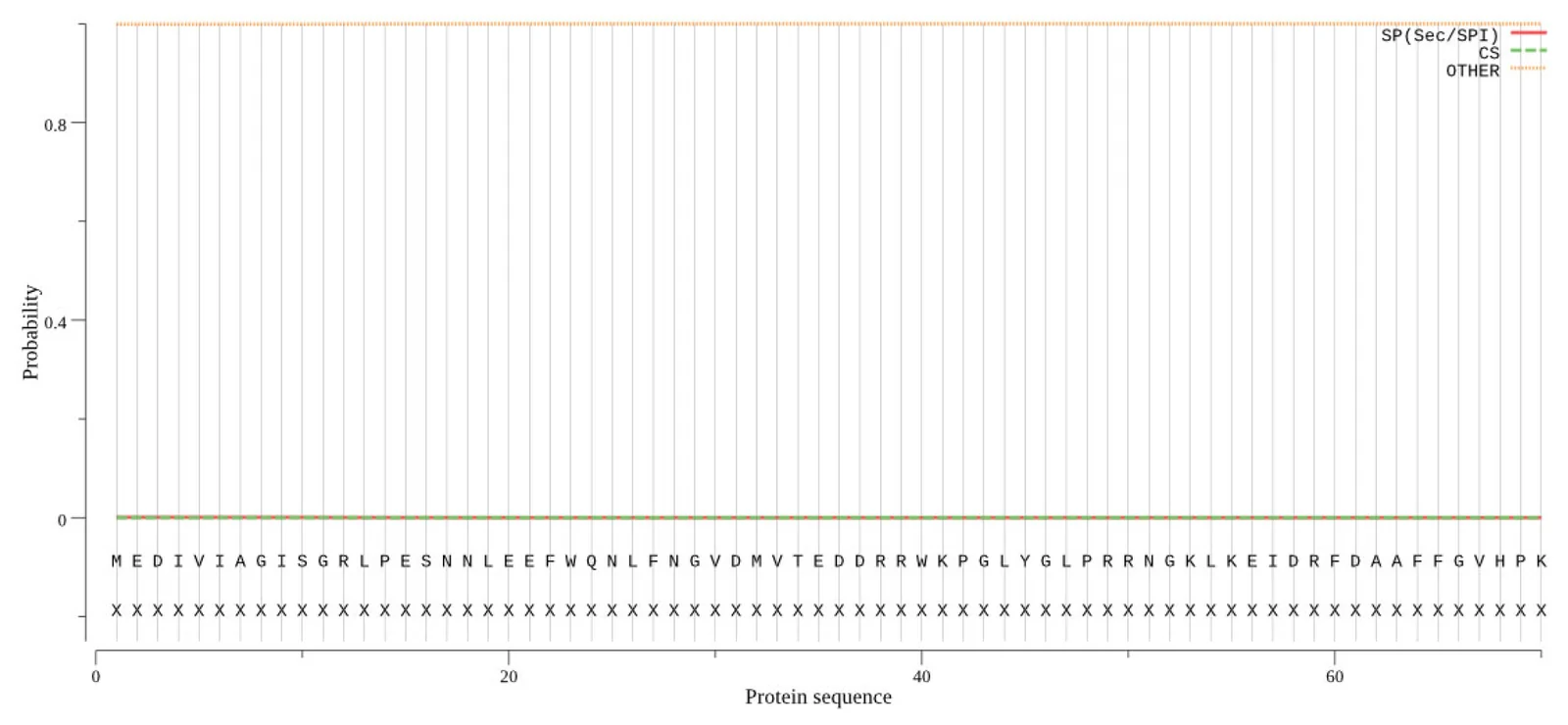
Signal peptide prediction of *FASN* protein.

**Figure 3:**
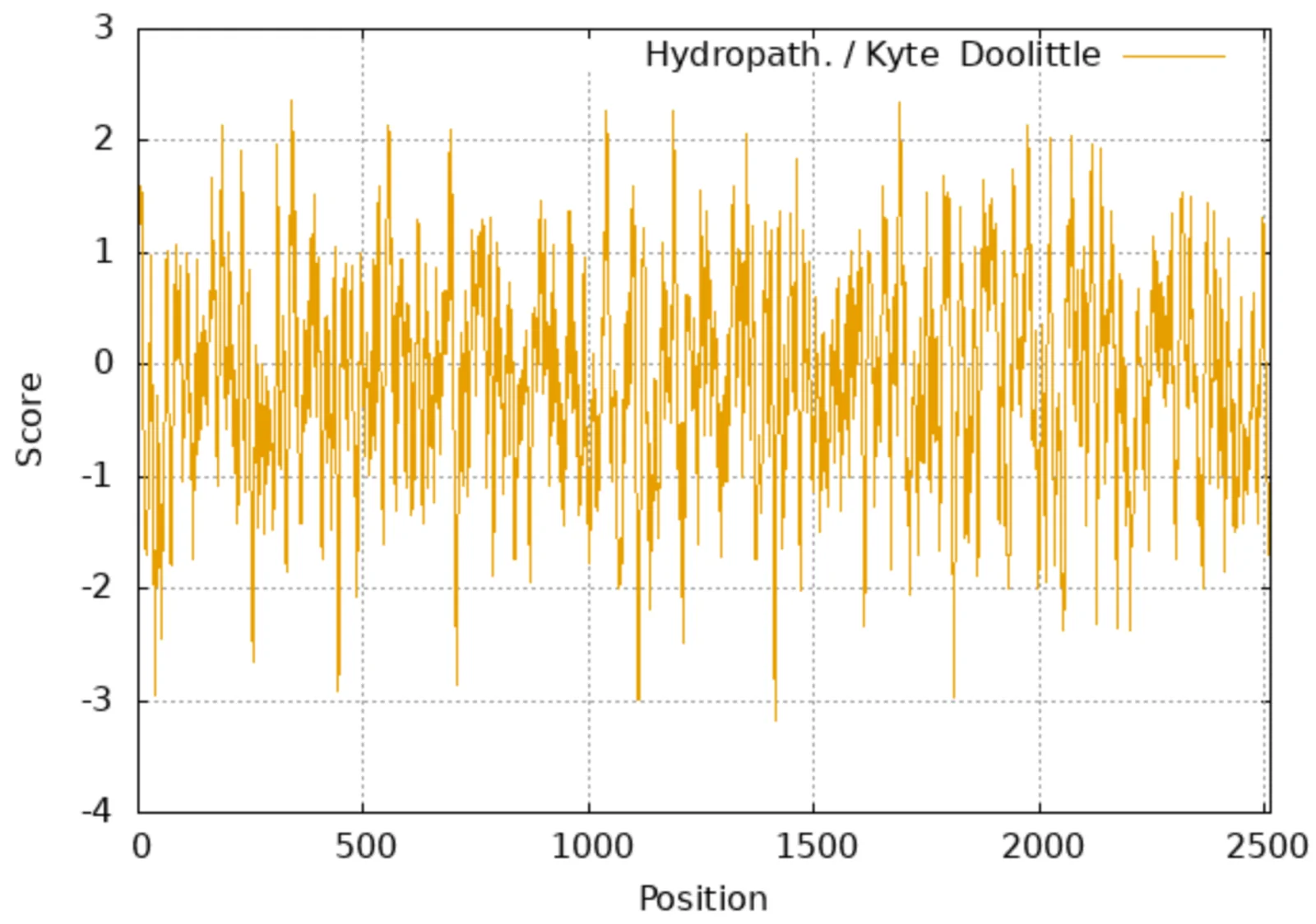
The hydrophilicity prediction of *FASN* protein.

**Figure 4:**
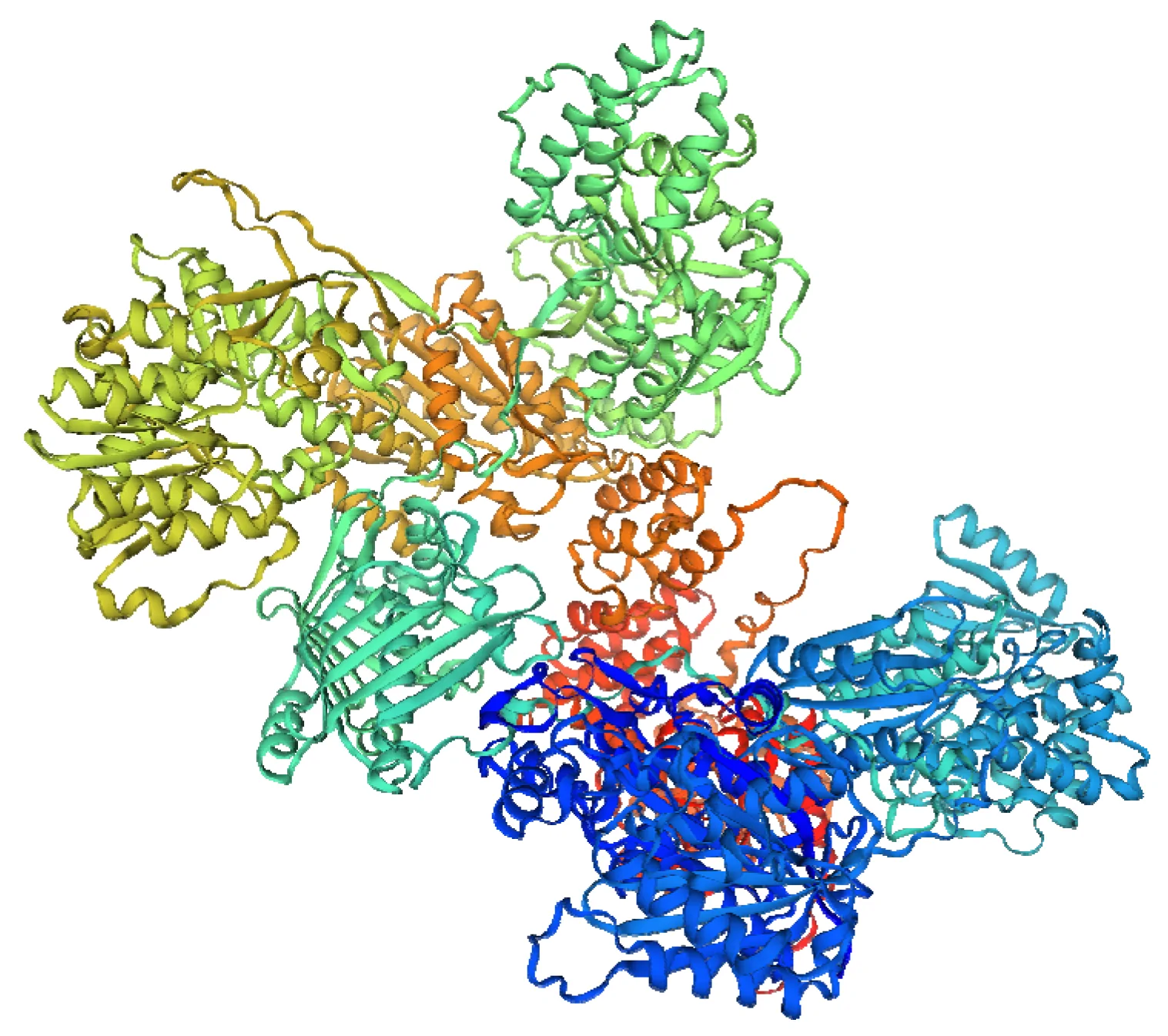
Tertiary structure prediction of *FASN* protein.

**Figure 5:**
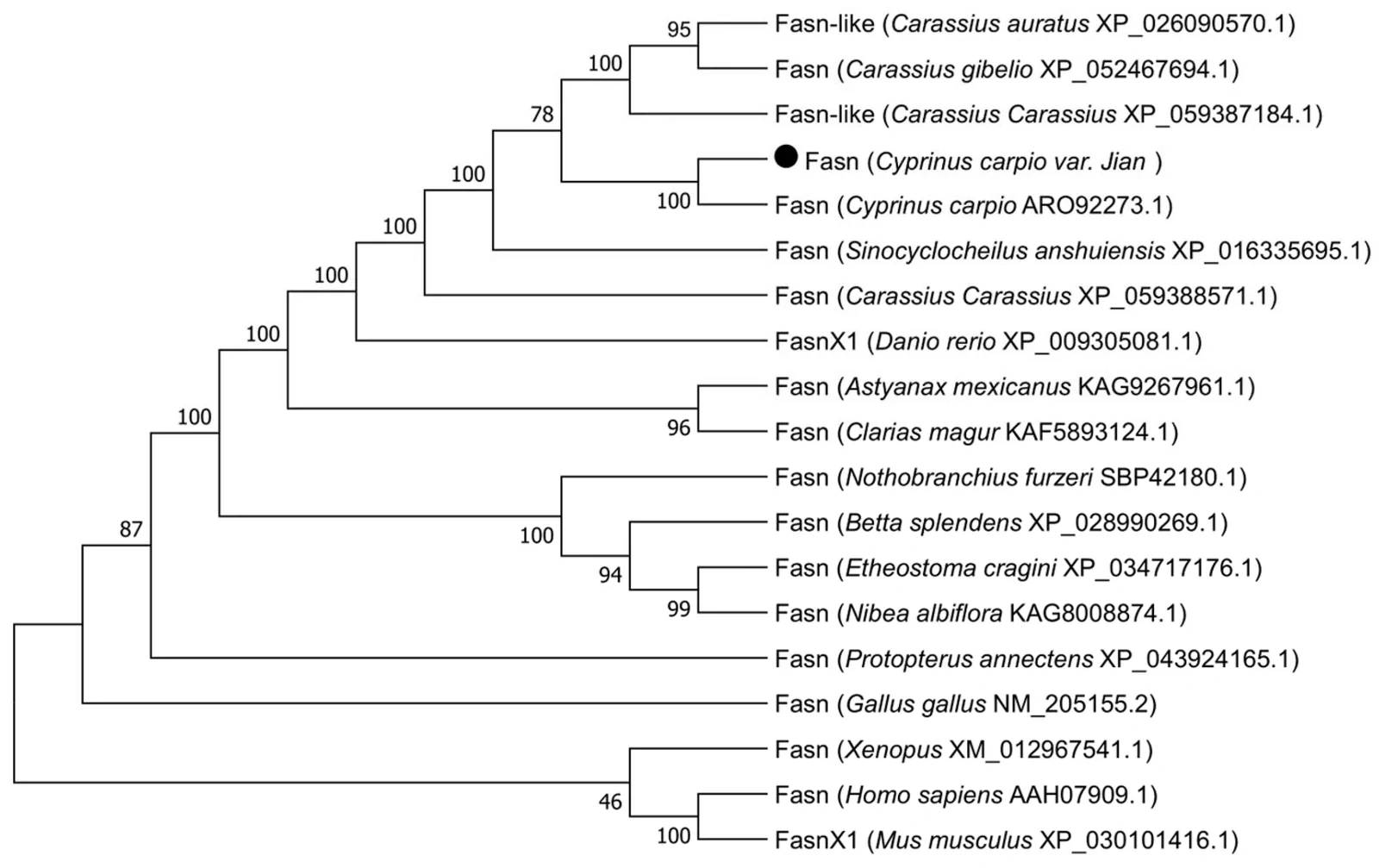
Phylogenetic analysis based on *FASN* amino acid sequence similarity.

**Figure 6:**
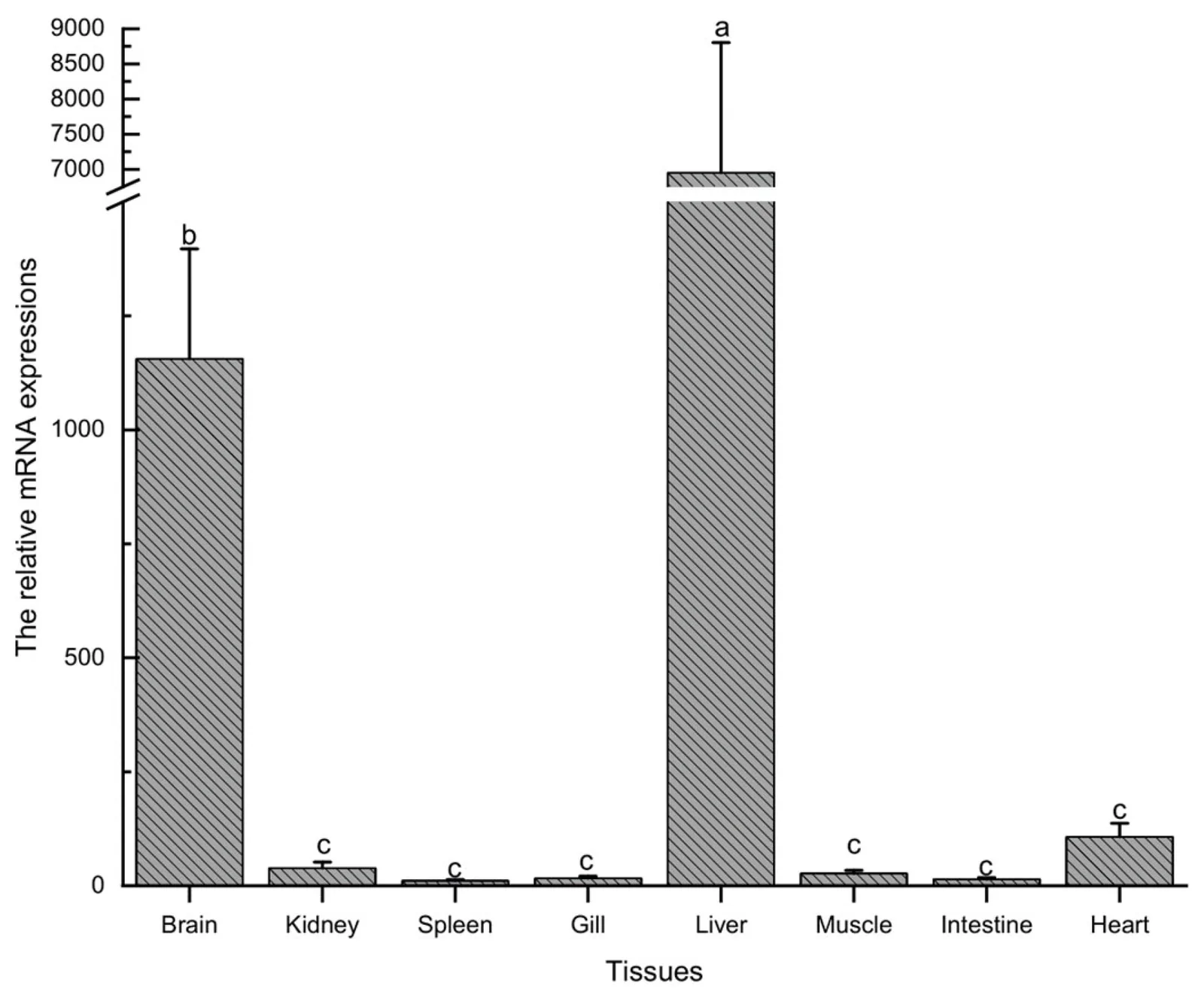
Tissue distribution profiles of *FASN* mRNA associated with hepatic lipid and redox metabolism in Jian carp. Note that the Y-axis is broken (marked by//) to accommodate the large dynamic range of mRNA expression values. Different lowercase letters indicate significant differences among tissues (*p* < 0.05).

**Figure 7:**
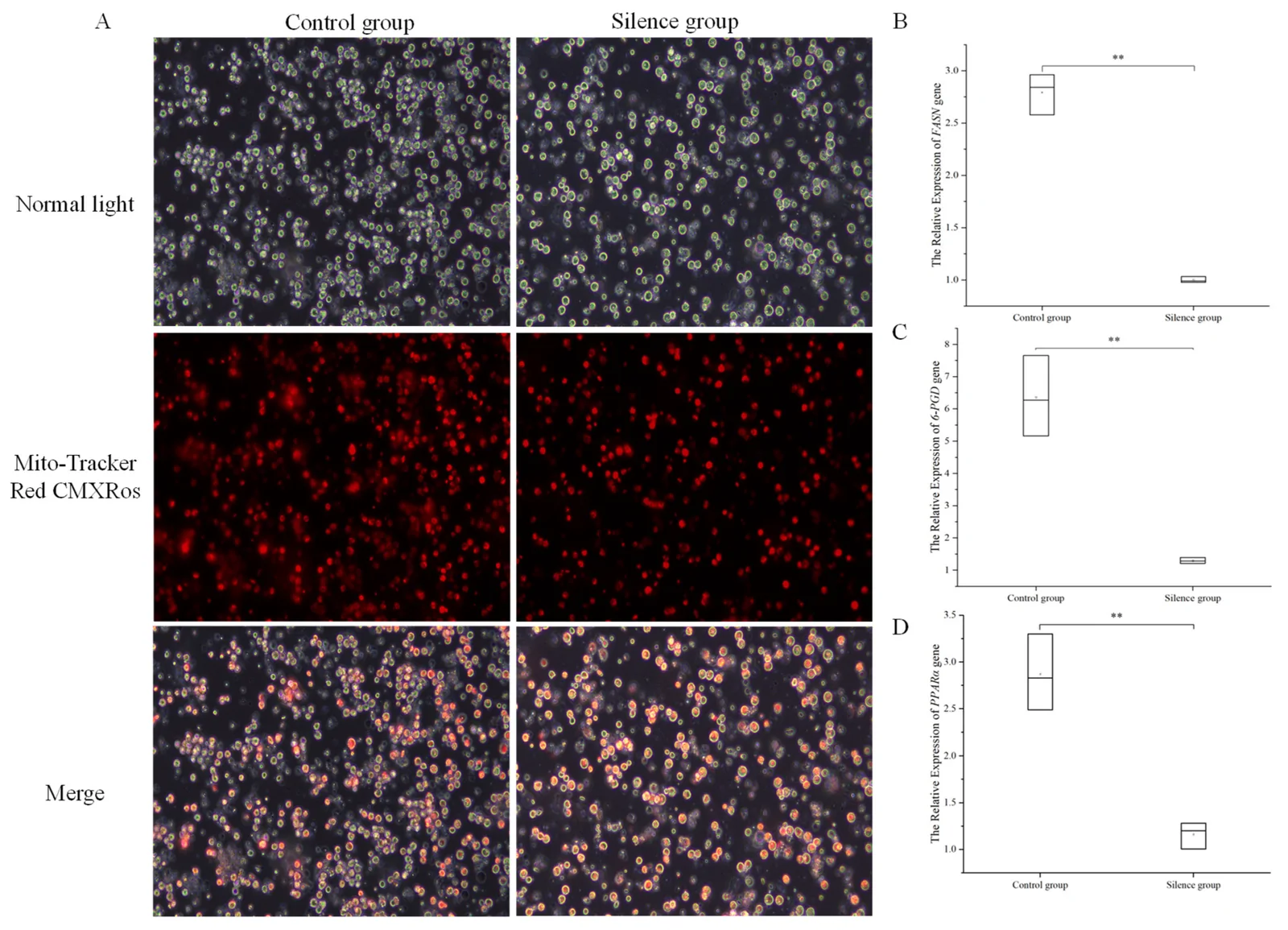
Effects of *FASN* knockdown on mitochondrial activity (**A**) and expression of lipid metabolism-related genes (*FASN*, *6-PGD*, *PPARα*) (**B**–**D**) in primary carp hepatocytes. siRNA-NC was used as the negative control group (control group). ** *p* < 0.01 compared with the control group.

**Figure 8:**
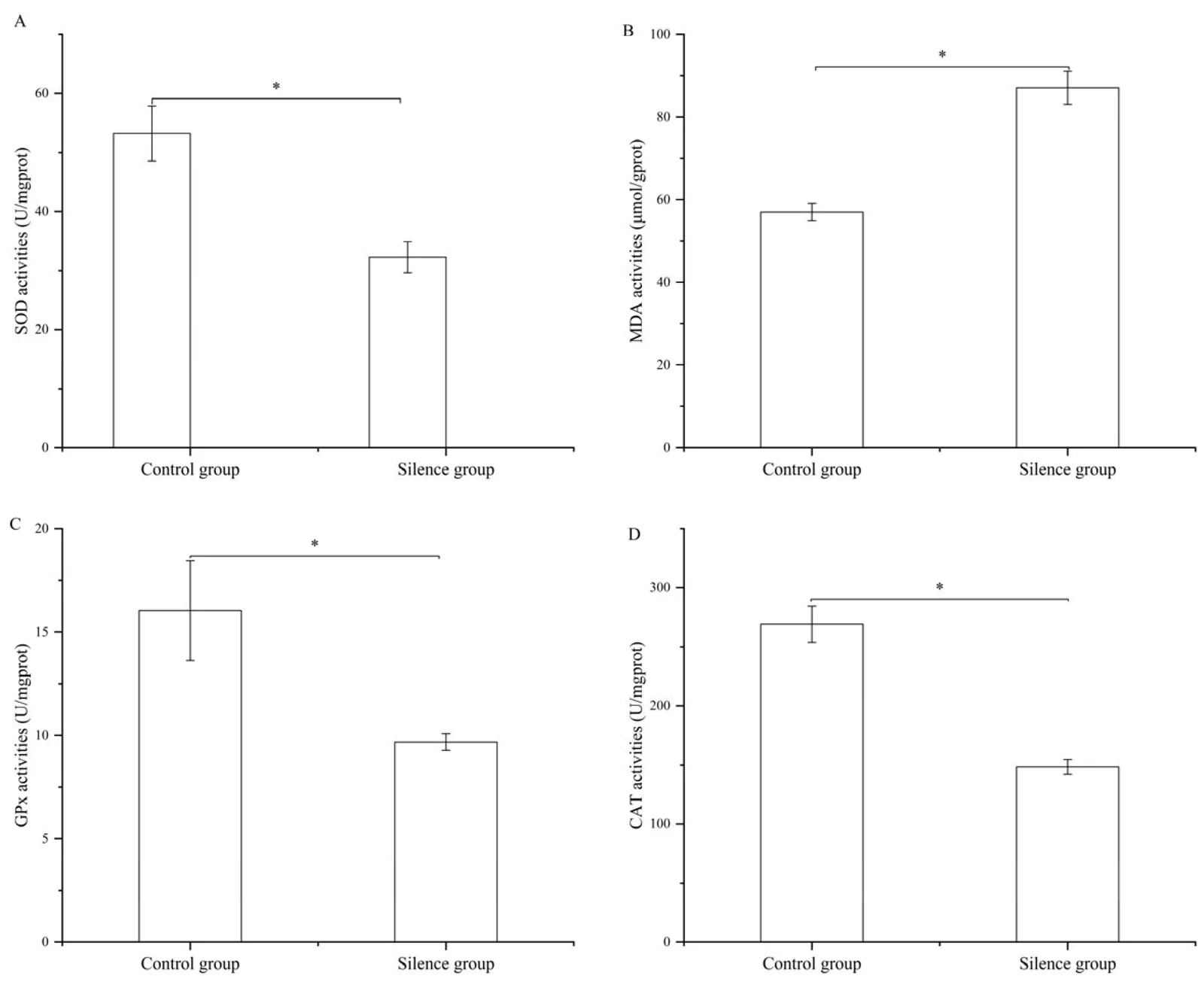
Effects of *FASN* knockdown on core antioxidant enzyme activities and lipid peroxidation marker MDA in primary Jian carp hepatocytes. siRNA-NC was used as the negative control group (control group). The activities of SOD (**A**), MDA (**B**), GPx (**C**), and CAT content (**D**) were measured in control and *FASN*-siRNA groups. Data are presented as mean ± SE (n = 3). * *p* < 0.05, compared with the control group.

**Figure 9:**
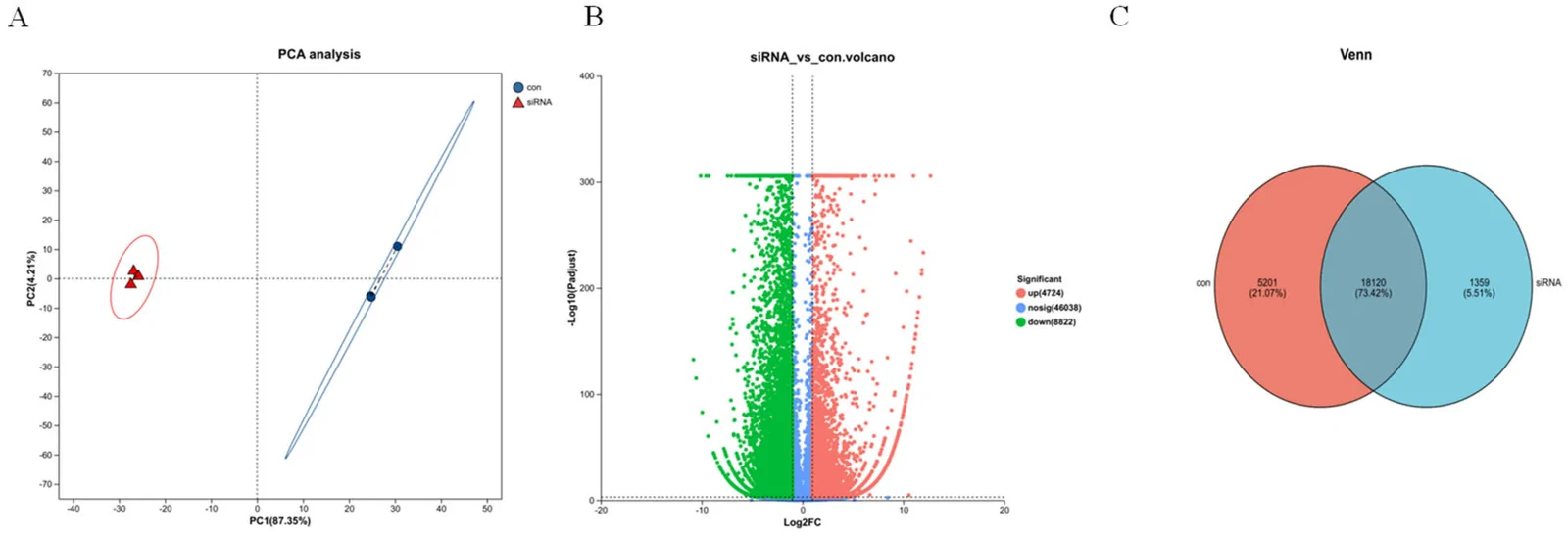
Transcriptomic analysis of primary hepatocytes following *FASN* knockdown. siRNA-NC was used as the negative control group (control group). (**A**) Principal component analysis (PCA) showing the separation between control (blue) and *FASN*-silenced (red) groups. (**B**) Volcano plot illustrating differentially expressed genes (DEGs) between the two groups. Red and blue dots represent significantly upregulated and downregulated genes, respectively (|log_2_FC| ≥ 1, FDR < 0.05). (**C**) Venn diagram displaying the number of uniquely and co-expressed genes in control and *FASN*-silenced groups.

**Figure 10:**
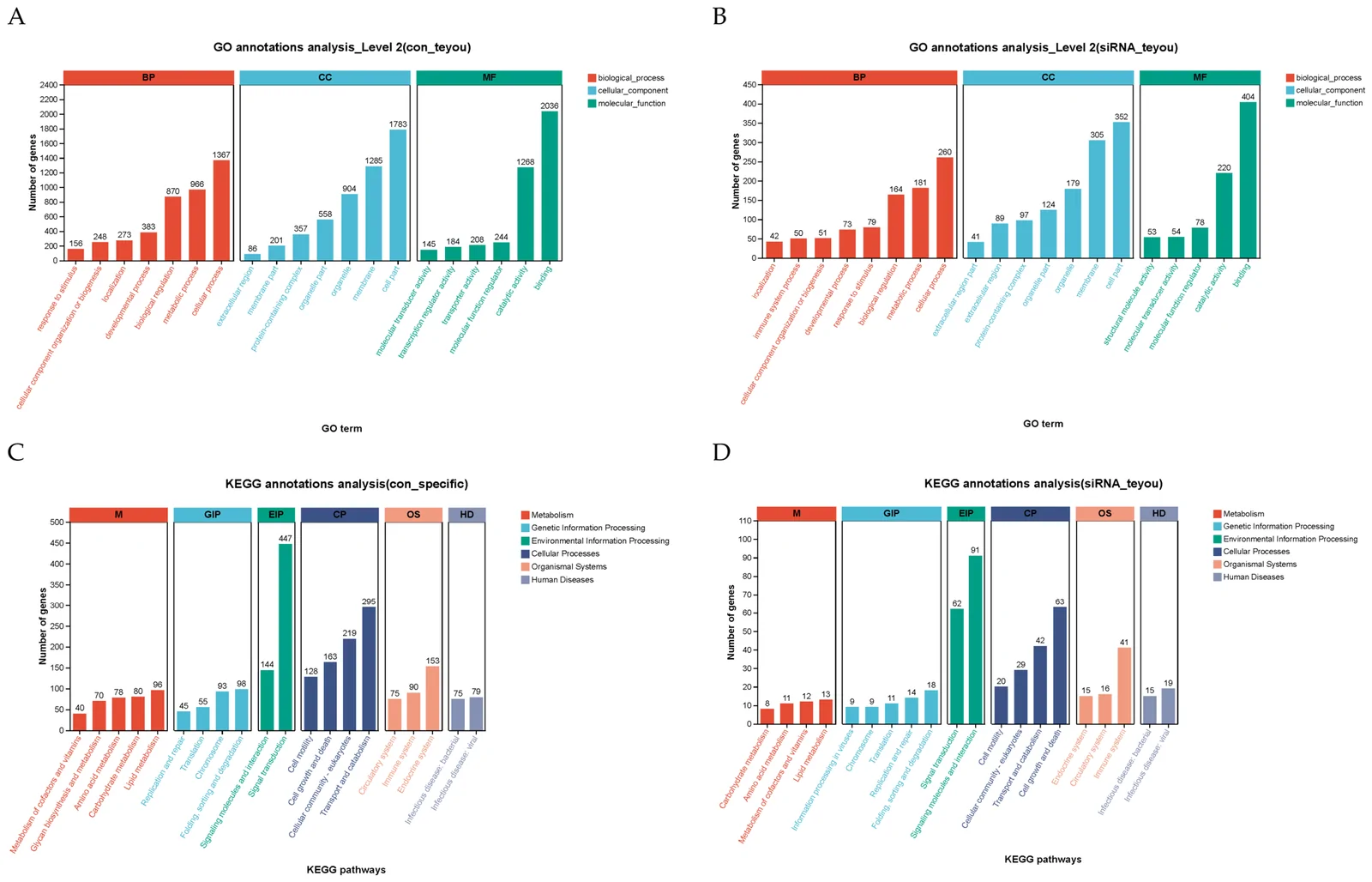
Functional enrichment analysis of group-specific genes in control and FASN-silenced hepatocytes. (**A**) GO enrichment analysis (Level 2) of genes uniquely expressed in the control group. (**B**) GO enrichment analysis (Level 2) of genes uniquely expressed in the *FASN*-silenced group. (**C**) KEGG pathway enrichment analysis of control-specific genes. (**D**) KEGG pathway enrichment analysis of siRNA-specific genes.

**Figure 11:**
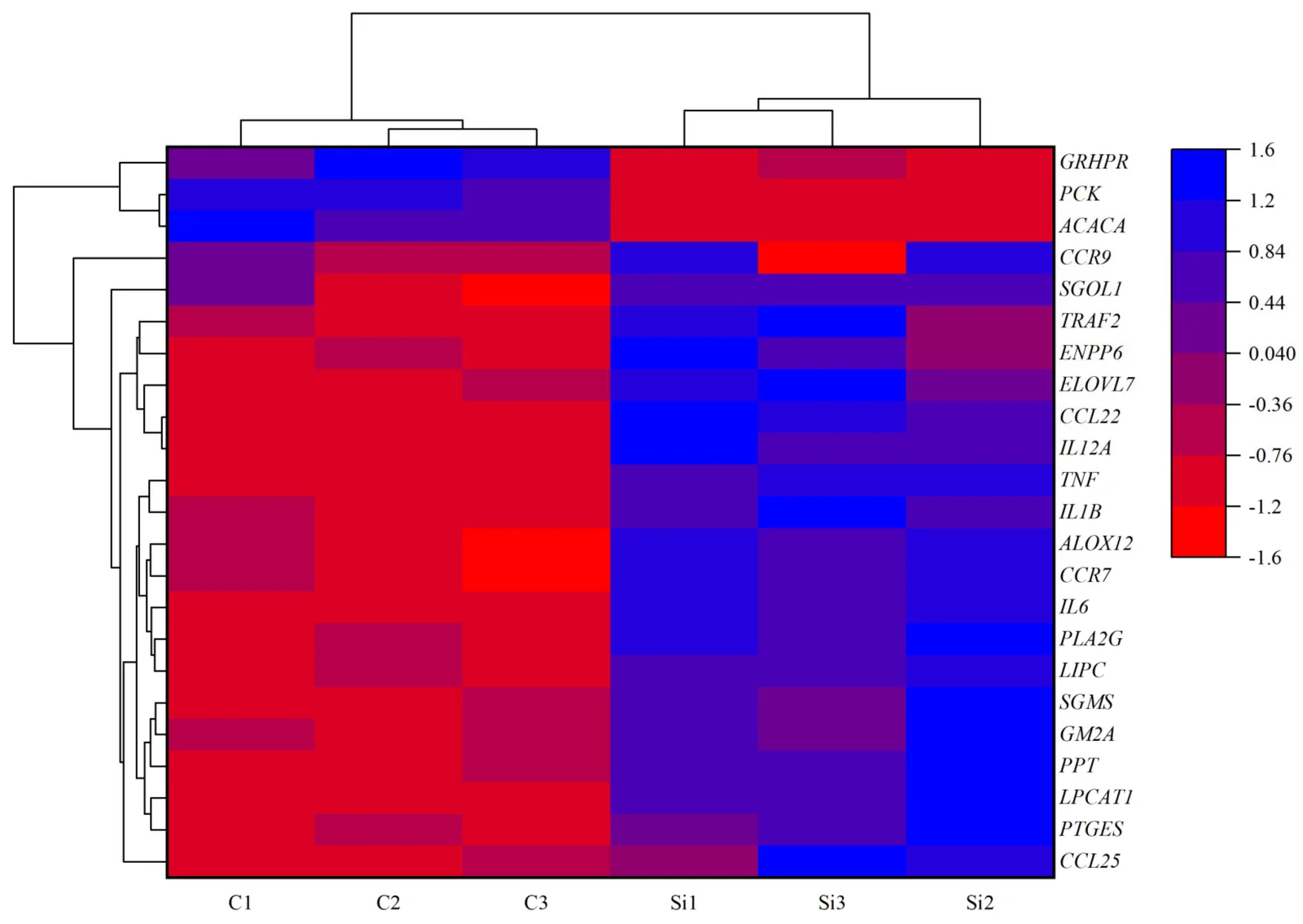
Heatmap clustering of differentially expressed genes in primary Jian carp hepatocytes following *FASN* silencing.

**Figure 12:**
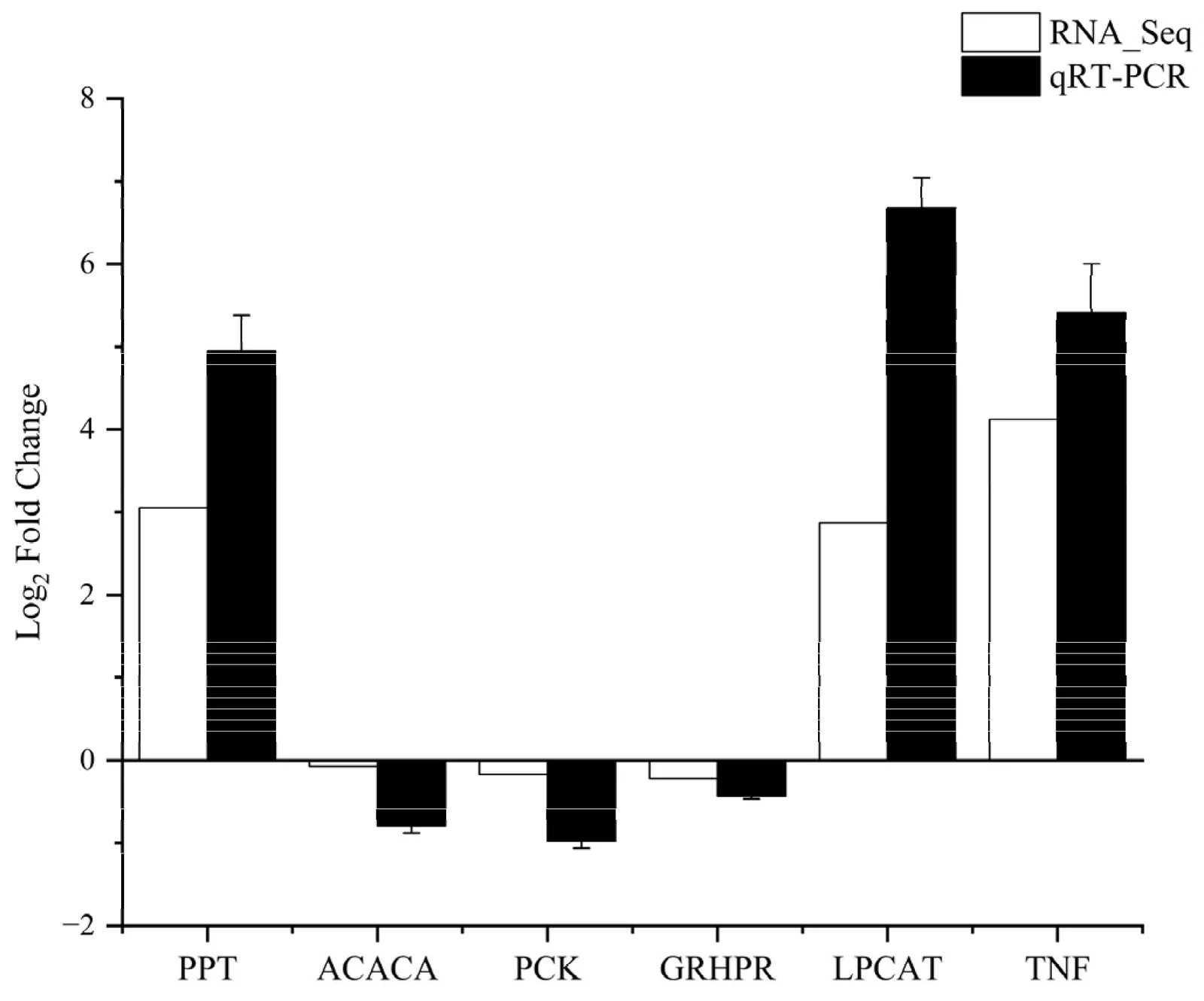
Validation of transcriptome expression profiles by qRT-PCR in *FASN* silencing hepatocytes of primary Jian carp hepatocytes.

**Table 1:** Sequences of primers used.

Gene Name	Forward Primer (5′ → 3′)	Reverse Primer (5′ → 3′)
*FASN*	AGACGGTAGCCTTCCTCACA	TTCACCATTCCCACCACTCC
*FASN*-siRNA	GCUCCGAUGUGGCUCUAAATT	UUUAGAGCCACAUCGGAGCTT
*6-PGD*	GCTCTGATGTGGCGAGGTGG	CGTAGAAGGACAGTGCAGTGGTAAA
*PPARα*	CGAGGATGGGATGCTGGTG	CGTCTGGGTGGTTCCGTCTGC
*PPT*	GAGACAGCTTCCTGCGCATA	TTCCAGTCTGCTAAACACCACA
*ACACA*	AACAGTGAGCTGACAGACGG	AGACCTTTATTGGTGATCTGGAG
*PCK*	ACCCCCACTGAAGCTGTACT	CAGCACTAGATGCATGCTGTTT
*GRHPR*	ATGCAAAAAGCCTCGAAGTGC	TCACACGCAAACTGACCAAAC
*LPCAT*	CGAACAGCCAACGGAACATC	CGAACAGCCAACGGAACATC
*TNF*	GGCGGAAATGGTGAGTTCAG	TGAGGTGAAGTGACGTTTAGCA
siRNA-NC	UUCUCCGAACGUGUCACGUTT	ACGUGACACGUUCGGAGAATT
β-actin	CGTGATGGACTCTGGTGATG	TCGGCTGTGGTGGTGAAG

**Table 2:** Fatty acid composition and content in between control and silence group (g/100 g).

Fatty Acid	Control Group	Silence Group
C14:0	0.037 ± 0.001	0.009 ± 0.009 **
C16:0	0.55 ± 0.02	0.21 ± 0.02 **
C16:1	0.062 ± 0.007	0.032 ± 0.001 *
C18:0	0.55 ± 0.02	0.17 ± 0.06 **
C18:1n9c	1.04 ± 0.12	0.48 ± 0.02 *
C18:2n6c (LA)	0.047 ± 0.006	0.027 ± 0.001
C20:1	0.14 ± 0.02	0.05 ± 0.001
C20:2	0.018 ± 0.002	0.0052 ± 0.0001 **
C22:0	0.035 ± 0.005	0.0075 ± 0.0008 **
C20:3n6	0.027 ± 0.006	0.012 ± 0.0003
C22:1n9	1.97 ± 0.15	0.66 ± 0.08 **
C20:4n6 (ARA)	0.055 ± 0.007	0.032 ± 0.0007
C22:2	0.024 ± 0.0004	0.011 ± 0.001 *
C24:1	0.066 ± 0.007	0.026 ± 0.001 *
C22:6n3 (DHA)	0.036 ± 0.002	0.027 ± 0.002 *
Σn-3	0.036 ± 0.002	0.027 ± 0.002 *
Σn-6	0.13 ± 0.01	0.071 ± 0.002 *
Σn-3/n-6	0.29 ± 0.05	0.38 ± 0.03
∑SFA	1.17 ± 0.009	0.39 ± 0.02 **
∑MUFA	3.28 ± 0.3	1.25 ± 0.07 *
∑PUFA	0.21 ± 0.01	0.11 ± 0.005 *

Note: siRNA-NC was used as the negative control group (control group). Data were expressed as mean ± SE from four biological replicates. Values represent fatty-acid mass (g) per 100 g wet-weight cell pellet. Asterisks indicate significant differences compared to the control group: * *p* < 0.05, ** *p* < 0.01. Abbreviations: ∑MUFA, monounsaturated fatty acid; ∑PUFA, polyunsaturated fatty acid; ∑SFA, saturated fatty acid.

**Table 3:** Summary of RNA-seq data.

Sample	Raw_Reads	Clean_Reads	Error_Rate	Q20 (%)	Q30 (%)	GC (%)
Control_1	36,698,238	36,329,216	0.0121	99.11	95.7	44.79
Control_2	43,853,688	43,426,602	0.0119	99.17	95.97	45
Control_3	42,724,546	42,359,180	0.012	99.16	95.94	44.62
Silence_1	37,790,322	37,526,198	0.0121	99.15	95.57	47.41
Silence_2	36,444,614	36,165,278	0.0121	99.16	95.69	46.48
Silence_3	45,126,508	44,811,728	0.0119	99.24	96.1	46.47

Note: siRNA-NC was used as the negative control group (control group).

**Table 4:** Key candidate genes specifically expressed in the silencing group.

Gene Symbol	Gene Name	Signaling Pathway
*CCL25*	*C-C motif chemokine ligand 25*	Chemokine signaling pathway
*IL12A*	*Interleukin 12A*	Cytokine–cytokine receptor interaction
*ELOVL7*	*Elongation of very long chain fatty acids protein 7*	Fatty acid metabolism
*PTGES*	*Prostaglandin E synthase*	Arachidonic acid metabolism
*LPCAT1*	*Lysophosphatidylcholine acyltransferase 1*	Glycerophospholipid metabolism
*PPT*	*Palmitoyl-protein thioesterase*	Protein palmitoylation/S-palmitoylation cycle
*ENPP6*	*Ectonucleotide pyrophosphatase/phosphodiesterase 6*	Sphingolipid metabolism
*LIPC*	*Lipase C*	Fatty acid metabolism
*ACACA*	*Acetyl-CoA carboxylase alpha*	Fatty acid biosynthesis
*GM2A*	*GM2 ganglioside activator*	Sphingolipid metabolism
*TRAF2*	*TNF receptor associated factor 2*	NF-kappa B signaling pathway
*PCK*	*Phosphoenolpyruvate carboxykinase*	Glycolysis/Gluconeogenesis
*IL1B*	*Interleukin 1 beta*	Cytokine–cytokine receptor interaction
*GRHPR*	*Glyoxylate reductase/hydroxypyruvate reductase*	Glyoxylate and dicarboxylate metabolism
*SGMS*	*Sphingomyelin synthase*	Sphingolipid metabolism
*TNF*	*Tumor necrosis factor*	Cytokine–cytokine receptor interaction
*CCR9*	*C-C motif chemokine receptor 9*	Chemokine signaling pathway
*CCR7*	*C-C motif chemokine receptor 7*	Chemokine signaling pathway
*CCL22*	*C-C motif chemokine ligand 22*	Chemokine signaling pathway
*PLA2G*	*Phospholipase A2 group*	Arachidonic acid metabolism
*ALOX12*	*Arachidonate 12-lipoxygenase*	Arachidonic acid metabolism
*SGOL1*	*Shugoshin 1*	Cell cycle
*IL6*	*Interleukin 6*	Cytokine–cytokine receptor interaction

## Data Availability

The original contributions presented in this study are included in the article and Supplementary Materials. The raw data supporting the conclusions of this article will be made available by the authors on request.

## Supplementary Materials

The following supporting information can be downloaded at: https://www.mdpi.com/article/10.3390/antiox15101237/s1.
