# Supplementary material for: Regulatory Functions of Fatty Acid Synthase on Redox Homeostasis and Lipid-Immune Crosstalk in Primary Hepatocytes of Cyprinus carpio var. Jian: A Comprehensive Evaluation of Antioxidant Capacity, Fatty Acid Profiles and Transcriptomic Signaling
Source: Antioxidants (Basel). 2026 Sep 24;15(10):1237. doi: 10.3390/antiox15101237 (PMC13700723; doi:10.3390/antiox15101237)
Supplement: Supplementary file 1 [file antioxidants-15-01237-s001.zip › antioxidants-4504085-supplementary.pdf]

1 GGGAGAAAGGCGAGAGTAATTTCTAGGGCTTGGCCAAAGGGGGGGAACACGAAACTCTCC

61 ATTCTGTACCGGGAGCCGACAAAGATTTAGAGGAATAAAGAAGTCAACTCTATCCCAAAG

121 GATCTGCGTGTAACACCGCGTTAAACTGTACCCACATCCAGAATCAAAGAAAATCTAACC

181 AGACATGGAGGATATTGTTATAGCAGGAATTTCAGGTCGTCTTCCCGAGTCAAACAATTT

1 M E D I V I A G I S G R L P E S N N L

PKS_KS

241 GGAAGAATTTTGGCAGAACCTTTTCAATGGAGTGGACATGGTCACAGAAGATGACAGGCG

20 E E F W Q N L F N G V D M V T E D D R R

301 GTGGAAACCAGGTCTGTATGGCCTTCCCCGAAGAAATGGAAAACTAAAAGAAATCGACAG

40 W K P G L Y G L P R R N G K L K E I D R

361 ATTTGATGCAGCCTTCTTTGGTGTGCATCCCAAACAGGCCCACACCATGGACCCACAGCT

60 F D A A F F G V H P K Q A H T M D P Q L

421 AAGGCTCATGCTAGAGATATCGTACGAGGCCATCGTCGATGGAGGCATAAACCCGGTGTC

80 R L M L E I S Y E A I V D G G I N P V S

481 TATGCGTGGCAGCAAGACTGGCGTTTATATTGGAGTAAGTGGCTCAGAGGCAGGAGAGGC

100 M R G S K T G V Y I G V S G S E A G E A

541 CTTCAGTAAAGACCCAGAAGAGCTGCTGGGATACAGCATGACAGGATGCCAGCGGGCCAT

120 F S K D P E E L L G Y S M T G C Q R A M

601 GTTTGCCAACCGCTTGTCTTACTTCTTTGATTTCAATGGTCCGAGTACAGCCATTGACAC

140 F A N R L S Y F F D F N G P S T A I D T

661 AGCATGCTCATCTAGTCTACTGGCTCTGGAAAATGCCTTCAATGCTATACGCCACGGCCA

160 A C S S S L L A L E N A F N A I R H G Q

721 ATGTGATGCAGCACTTGTGGGCGGAGTCAACCTGCTGCTCAAGCCCAACACCTCAGTGCA

180 C D A A L V G G V N L L L K P N T S V Q

781 GTTTATGAAACTGGGCATGCTCAGTCCTGAAGGAGCATGCAAGTCATTTGATGCCTCAGG

200 F M K L G M L S P E G A C K S F D A S G

841 AAATGGCTATTGTCGCTCAGAGGCTGCCGTCGCAGTACTCTTGACCAAGAAGTCAATGGC

220 N G Y C R S E A A V A V L L T K K S M A

901 CAAGAGAATCTACGCCACTGTTCTCAACACAGGCAATAACACAGACGGATACAAAGAGCA

240 K R I Y A T V L N T G N N T D G Y K E Q

961 AGGGGTCACATTCCCATCAGGTGAGATGCAGCAGCGCCTGGTCCGTTCCCTTTACCAGGA

260 G V T F P S G E M Q Q R L V R S L Y Q E

1021 GGCCAACATATCACCTGAGCAGGTGGAGTACATCGAGGCCCACGGCACCGGTACTAAGGT

280 A N I S P E Q V E Y I E A H G T G T K V

1081 TGGGGATCCTCAGGAAGTGAATGGCATTGTGAGTGTATTCTGTCAGTCACAGAGGGATCC

300 G D P Q E V N G I V S V F C Q S Q R D P

1141 TCTTCTGATTGGCTCAACCAAATCCAACATGGGACACCCAGAGCCTGCCTCTGGATTGGC

320 L L I G S T K S N M G H P E P A S G L A

1201 AGCTCTTGCCAAGGTGGTTTTGTCTCTGGAACATGGCGTCTGGGCTCCCAACATCCATTT

340 A L A K V V L S L E H G V W A P N I H F

1261 CCACGATCCAAACCCTGACATTCCTGCTCTGACAGACGGCCGGGTGCGTGTGGTGTCGGA

360 H D P N P D I P A L T D G R V R V V S E

1321 GCCCATACCTGTCCGTGGTGGCATCGTGGGCATCAACTCATTTGGCTTCGGAGGCTCAAA

380 P I P V R G G I V G I N S F G F G G S N

1381 TGTCCATATTATCCTACGTCCACATGATAAAAAAACACTGGCTCAGTCTGTACAACCTTC

400 V H I I L R P H D K K T L A Q S V Q P S

1441 AGTCCCCAGACTTCTGCAGGCCTCTGGTCGCACCGAGGAGGCGGTCACTGCCCTCCTCAA

420 V P R L L Q A S G R T E E A V T A L L N

1501 CAACGCCCAGCAGCATAAGGATAACCCAAGCTACCTGTCTCTTCTGAATGATGTGTCTGG

440 N A Q Q H K D N P S Y L S L L N D V S G

1561 AGTTCCAACGGCAGGCATGCCGTACAGGGGCTATGCACTGATCGGAGCACAGGGAGAGCT

460 V P T A G M P Y R G Y A L I G A Q G E L

1621 TACAGAGGTTCAGCAAGCCCAGCCCACACCCAGACCGCTCTGGTACATTTGTTCAGGTAT

480 T E V Q Q A Q P T P R P L W Y I C S G M

PKS_AT

1681 GGGCACACAGTGGGCTGGAATGGGCCGTACCCTGATGCAGCTGCCAGAATTCCGGGAGTC

500 G T Q W A G M G R T L M Q L P E F R E S

1741 GATCCTGCGCTCCGATGTGGCTCTAAAGCACACTGGGTTGTGTGTGTCTCGTTTGCTCAT

520 I L R S D V A L K H T G L C V S R L L M

1801 GGATGCTGATGAGAGCACTTTTGAAGACACTGTGCACGCATTTGTTGGACTTGCAGCCAT

540 D A D E S T F E D T V H A F V G L A A I

1861 CCAGGTCGCCCAGATTGACATGTTGCAGAGGATGGGTCTTCAGCCTGATGGGATCGTGGG

560 Q V A Q I D M L Q R M G L Q P D G I V G

1921 CCACTCTGTGGGAGAGCTAGCGTGTGGTTATGCCGACGGCTCCCTGAGCCACAGTGAGAC

580 H S V G E L A C G Y A D G S L S H S E T

1981 CATTCTAGCTGCTTACTGGAGAGGACGCTGTATTAAAGAGGCCAACCTGCCCCCGGGTGG

600 I L A A Y W R G R C I K E A N L P P G G

2041 CATGGCTGCAGTGGGCTTAACGTGGGAAGAATGCAAAGCTCAGTGTCCACAGGGTGTCGT

620 M A A V G L T W E E C K A Q C P Q G V V

2101 TCCTGCTTGTCACAATGCTGAGGACACAGTGACCATCTCAGGCCCTCAGGACTCTGTCAG

640 P A C H N A E D T V T I S G P Q D S V S

2161 CAAGTTTGTTGCCCAGCTGAAAGAGAGCGGTGTGTTTGCCAAAGAAGTGCGCAGTGCCGG

660 K F V A Q L K E S G V F A K E V R S A G

2221 TGTGGCTTTCCACTCCTATTACATGGCCTCTATTGCTCCAGCCCTTCTGTCTGCACTGCA

680 V A F H S Y Y M A S I A P A L L S A L Q

2281 GAAGGTAATAAAGAACCCAAGGCCTCGTTCATCACGTTGGATCAGCACGTCCATCCCGCA

700 K V I K N P R P R S S R W I S T S I P Q

2341 GGCAGAATGGGAGAGCCCACTGGCACTCTACTCATCTGCAGAGTACCATGTCAATAACTT

720 A E W E S P L A L Y S S A E Y H V N N L

2401 GGTCAGCCCTGTGCTCTTTCAGGAGGGACTAAGCTTTGTGCCAGATAATGCTGTGGCTGT

740 V S P V L F Q E G L S F V P D N A V A V

2461 AGAAATTGCTCCACATGCTTTGCTGCAGGCCATCCTGAAACGGAGCCTGAAACCTACCTG

760 E I A P H A L L Q A I L K R S L K P T C

2521 CTCCATCCTTCCCCTAATGAAAAGAGGACATGCCAACAATCTGGAGTTCTTCCTTTCTCA

780 S I L P L M K R G H A N N L E F F L S H

2581 TGTGGGCAAGATCTACATGAATGGAATTAACGTGGACAGTAATAAATTGTATCCAGCTGT

800 V G K I Y M N G I N V D S N K L Y P A V

2641 TGAATACCCTGTTCCGACGGGGACCCCTCTCATCTCCCCTCACATTCAGTGGGATCATTC

820 E Y P V P T G T P L I S P H I Q W D H S

PKS_DH

2701 CCAGGTCTGGGACGTGCCCAAGGTGGAGGACTTCCCTGCTGGCTCAGGGGGATCCACCTC

840 Q V W D V P K V E D F P A G S G G S T S

2761 AGCCACTGTGTACAATATCGACATGAATCCAGAGTCTCCTGATTACTACATGATTGGCCA

860 A T V Y N I D M N P E S P D Y Y M I G H

2821 CTGCATTGATGGTCGGGTGCTGTACCCAGCCACAGGATACCTGGTTCTGGCCTGGAGGAC

880 C I D G R V L Y P A T G Y L V L A W R T

2881 TCTGATGAGATCTCTTGGTGCAGTCATGGAGCACACACCTGTTACATTTGAAGACGTCAC

900 L M R S L G A V M E H T P V T F E D V T

2941 TATCCATAGAGCCACCATCCTACCCAAGACTGGGTCTGTCCAGCTGGAGGTGCGACACAT

920 I H R A T I L P K T G S V Q L E V R H M

3001 GCCCGCCACTAACCGTTTTGAGGTGTCAGAGAATGGAAATCTGGCCGTCAGTGGTAAGGT

940 P A T N R F E V S E N G N L A V S G K V

3061 GAGTGTCTTGGAGGATGCTGGACTTGATGCTTTCCATGCTGAGCTGAATAAACCAATGGC

960 S V L E D A G L D A F H A E L N K P M A

3121 TGTAGACAAGGAAGATTCTAAACTGCTTTTGAAAGCCGGGGACATCTACAAAGAGCTGCG

980 V D K E D S K L L L K A G D I Y K E L R

3181 TCTGCGTGGATATGACTACGGCAAGACTTTCCAGGGAATCCTGGAGTCTAACAATGCTGG

1000 L R G Y D Y G K T F Q G I L E S N N A G

3241 TGACTGTGGAAAGCTTCACTGGACAGGTAACTGGGTGACGTTTCTAGACACCATGCTGCA

1020 D C G K L H W T G N W V T F L D T M L Q

3301 AATGATCGTAGTGGGACTGCCTGGTCGTAGCTTGAGACTGCCCACTCGCATACGCTCTGT

1040 M I V V G L P G R S L R L P T R I R S V

3361 GTATGTGGACCCCAAACTTCATGAGGAGAGGCTAAACGAGTATGCAGATGGCCAGAAAGC

1060 Y V D P K L H E E R L N E Y A D G Q K A

3421 TGTGGACGTTCATGTCAACCGTTGCCTTGACAACATAACAGCTGGAGGAGTTCAGATCTG

1080 V D V H V N R C L D N I T A G G V Q I C

3481 TGGGCTTCATGCCACTGTAGCACCCCGTCGCCAACAGCAGCAGACTCCACCCACTTTGGA

1100 G L H A T V A P R R Q Q Q Q T P P T L E

3541 GGAGTTTGTCTTTGTTCCCTATGTGGACTCAGATTGCCTGAGAACCAATGAAAAACTTGG

1120 E F V F V P Y V D S D C L R T N E K L G

3601 AGACCAACTGAGACATTGCAAAGGTCTAGTACAGCGTCTGCAGAGGAAGTTGGCCAATCA

1140 D Q L R H C K G L V Q R L Q R K L A N Q

3661 GGGAGTGAAGATCTCCATCCCTGGGTTAGAAGGTGCGGCTGAGGGCCAGCTGATTGGAGC

1160 G V K I S I P G L E G A A E G Q L I G A

3721 GGAGGCAGAGAAAGGCCTGTTGCGGTTGCTCTCTGTGCTATGCGGGCTGGAACTGAACGG

1180 E A E K G L L R L L S V L C G L E L N G

3781 AAACCTGCGCTCTGAGCTGGAACAGACGGTGGAGAAGGAGAGAGACTGTCTGCTGCAGGA

1200 N L R S E L E Q T V E K E R D C L L Q D

3841 CCCTCTGCTCAATGGCCTGCTTGACTCCCAGGCGCTACGTCACTGCTTGGACACGGCCCT

1220 P L L N G L L D S Q A L R H C L D T A L

3901 TGAGAACAGCACTCCTGGAAAACTCAAAGTTGTGGAGGCCCTAGCCGCCGACGGTCGTGT

1240 E N S T P G K L K V V E A L A A D G R V
3961 GTTTTCTCGAACTGTTGGTCTCCTCAATATCCAGCCGATGTTGCGTCTCGACTACACTGT
1260 F S R T V G L L N I Q P M L R L D Y T V
4021 TTCAGACGTCTCCATTGAAAAGCTTTCAGCACACCAGAGCTCCTTGGAAGAGCAGGGCAT
1280 S D V S I E K L S A H Q S S L E E Q G I
4081 CTCATCTGCTCAGTGGGACCCTCTTCAGGGCCCAGCAACAGGTGGTCTGGATGGGGCTGA
1300 S S A Q W D P L Q G P A T G G L D G A D
4141 CCTGGTTGTGTGTAACTGTGCAACAGGGCACATGGCCAACCCTGCACTTCTGATAGAGAA
1320 L V V C N C A T G H M A N P A L L I E N
4201 CCTGACTTCAGCTGCGAGAGAAGGCGGTTTTGTTTTGATCCACACCCTTCTGAGAGGAGA
1340 L T S A A R E G G F V L I H T L L R G D
4261 CACTCTGGGAGAGACGGTAGCCTTCCTCACATCTCAAAACAACCAGGAGGGTTTGTTGAC
1360 T L G E T V A F L T S Q N N Q E G L L T
4321 ACAGACTGAATGGGAGGAGCTCCTCCAAAAGGCCTCTTTAAATGTGGTCATGCTGAGAAA
1380 Q T E W E E L L Q K A S L N V V M L R K
4381 GTCCTACTACGGCTCTGCTCTCTTCCTCTGCCACCGGCACCGACAGTCCCCTCAGGATCA
1400 S Y Y G S A L F L C H R H R Q S P Q D Q
4441 ACCTATCCTTATTTTTGTAGACCCTACTGACTACAAATGGGTGGAGCATCTAAAGAGCAC
1420 P I L I F V D P T D Y K W V E H L K S T
4501 TTTAGCCGAGTCATCTGATATCCCAGTCTGGCTCATTGCCACTAACGGTCATAATGGAGT
1440 L A E S S D I P V W L I A T N G H N G V
4561 GGTGGGAATGGTGAACTGCCTCAGGCAGGAGCCTGGAGGCAATCGTATACGGTGTGCATT
1460 V G M V N C L R Q E P G G N R I R C A F
4621 TGTTTCTAATCTAAGTAAGGGTGCTGAAGTCCCCTCCCTTGTTCCTGCCCAAAAAGAGAT
1480 V S N L S K G A E V P S L V P A Q K E M
4681 GAAGACATTACTAAAGAAGGATCTGGTCATGAACGTGTACCGTGATGGACATTGGGGCAT
1500 K T L L K K D L V M N V Y R D G H W G I
4741 TTTTAGACACCAGTTGTTAACACAAGATCTTAGTGAGGAGTTGACAGAGCAGGCGTATGT
1520 F R H Q L L T Q D L S E E L T E Q A Y V
4801 GAATGTACTGACTCGCGGTGATCTCTCATCGCTGCGATGGATTGCTTCCCCGCTGCGTCA
1540 N V L T R G D L S S L R W I A S P L R H

PKS_ER

4861 TTTTGTCCCTTCCAATCCCAACGTGCAGCTCTGCCGTGTTTACTACTCCTCCCTCAACTT
1560 F V P S N P N V Q L C R V Y Y S S L N F
4921 CAGAGACATCATGCTGGCCACCGGCAAACTGCCTCCAGATGCCATCCCTGGAGACATAGC
1580 R D I M L A T G K L P P D A I P G D I A
4981 GCTGCAGCAGTGCATGCTGGGAATGGAGTTTTCAGGACGAGACCCCAGTGGGCGACGTGT
1600 L Q Q C M L G M E F S G R D P S G R R V
5041 AATGGGTTTGCTGCCAGCTAAAGGTCTGGCCACATGTGTGGATGCAGACAAGCGCTTCCT
1620 M G L L P A K G L A T C V D A D K R F L
5101 TTGGGATGTTCCATCTAGCTGGACACTGGAGCAGGCCGCCTCTGTTCCTGTGGTGTACGC
1640 W D V P S S W T L E Q A A S V P V V Y A
5161 CACCGCCTATTATTCCCTAGTGGTGCGAGGGCGGCTGAGGCCTGGTGAAAGTGTGCTGAT
1660 T A Y Y S L V V R G R L R P G E S V L I

5221 CCATTCAGGCTCTGGAGGGGTTGGACAGGCCGCTATTGCTATTGCACTTAGTATGCGCTG
1680 H S G S G G V G Q A A I A I A L S M R C
5281 CCGAGTCTTCACAACTGTGGGTTCTGGAGAGAAGAAGGCGTATCTGCAAGAAAGATTTCC
1700 R V F T T V G S G E K K A Y L Q E R F P
5341 TCAGCTTACGGCAGAATCTTTTGCTAATTCTAGAGATGCCTCATTTGAACATCATGTAAT
1720 Q L T A E S F A N S R D A S F E H H V M
5401 GCTCCAAACTCAAGGCAAAGGAGTTGATTTGGTATTAAACTCCCTTGCTGAAGAGAAGCT
1740 L Q T Q G K G V D L V L N S L A E E K L
5461 GCAGGCCAGCTTGCGTTGTTTGGCCAGACATGGTCGTTTCCTGGAGATCGGCAAATATGA
1760 Q A S L R C L A R H G R F L E I G K Y D
5521 CCTTTCCAACAACACACCACTAGGTATGGCTCTGTTCCTGAAGAACGTGGCCTTCCACGG
1780 L S N N T P L G M A L F L K N V A F H G
5581 CATCCTGCTGGATGCTTTGTTTGAGGAGGGGAACAGGGAATGGGAGGAGGTATCTGAGCT
1800 I L L D A L F E E G N R E W E E V S E L
5641 TCTGAAGAAGGGAATCGCTGGTGGTGTAGTTCAGCCATTACGTACCACTGTCTTTGAGAG
1820 L K K G I A G G V V Q P L R T T V F E R
5701 GAACCAGGTGGAGGACGCCTTCCGTTACATGGCTCAGGGCAAACACATCGGCAAAGTCCT
1840 N Q V E D A F R Y M A Q G K H I G K V L
5761 GCTGCAGGTACGTTCAGAGGAGAAAAGCACCACTGTTCCTACAGTGTCTTCTCTTTCTAT
1860 L Q V R S E E K S T T V P T V S S L S I
5821 TCCCGCTATCTGCCGTACCTTCTTCCCTGCCTCTCTCTCGTACATCATCACTGGAGGCTT
1880 P A I C R T F F P A S L S Y I I T G G L

PKS_KR

5881 GGGTGGTTTCGGGTTGGAGTTGGCCCAGTGGCTTACTGAGAGAGGTGCTCGTAAGCTGGT
1900 G G F G L E L A Q W L T E R G A R K L V
5941 GCTGACGTCACGTTCTGGCATCCGCAACGGTTATCAAGCCAAGCGTATTCGAGAGTGGCA
1920 L T S R S G I R N G Y Q A K R I R E W Q
6001 GGCCATGGGCATTCAGGTTCTTGTGTCCACTAGTGATGTCAGCTCCATGGCAGGGACGGA
1940 A M G I Q V L V S T S D V S S M A G T E
6061 GCGTCTCATCACCGAGGCCTGCCGCTTGGGTCCAGTTGGTGGCATCTTCCACCTTGCCAT
1960 R L I T E A C R L G P V G G I F H L A M
6121 GGTCCTTAAAGACGGCATGCTGGAGAATCTGACCCCACAGCACTTCATTGATGTCAACAG
1980 V L K D G M L E N L T P Q H F I D V N R
6181 ACCCAAATACGATGGAACCATTCATCTTGACAGTGTGACTAGGCAGAAGTGCCCAGAACT
2000 P K Y D G T I H L D S V T R Q K C P E L
6241 TCAGCACTTTGTGGTGTTCTCATCGGTCAGCTGTGGCCGTGGTAACGCTGGCCAAAGCAA
2020 Q H F V V F S S V S C G R G N A G Q S N
6301 CTACGGCTTCGCCAACTCCACCATGGAGCGAGTGTGTGAGCAGCGCCGCCATGACAGCCT
2040 Y G F A N S T M E R V C E Q R R H D S L
6361 GCCGGGCCTGGCCATCCAGTGGGGTGCCATTGGAGATGTGGGTGTTGTCTTGGAGACCAT
2060 P G L A I Q W G A I G D V G V V L E T M
6421 GGGTGGCAATGATGCAGTGATTGGTGGTACGTTGCCCCAACGCATGTCTTCCTGTTTGGA
2080 G G N D A V I G G T L P Q R M S S C L E
6481 AGTCCTGGATAGGTTCCTGTGCCAGCGGAGGCCTGTAATGTCCAGCTTTGTGTTGGCGGA

2100 V L D R F L C Q R R P V M S S F V L A E
6541 GAAAGTGGTGGTCGCTAAAGGAGAAGGAAGTGGACAGAGGGACCTGGTGGAGGCTGTGGC
2120 K V V V A K G E G S G Q R D L V E A V A
6601 TCATATTCTGGGTGTGCGTGATGTGAACAGTCTGAATGCCGATGCCTCATTGGCTGATCT
2140 H I L G V R D V N S L N A D A S L A D L
6661 GGGTCTCGATTCGCTGATGGGTGTTGAGGTTAGGCAGACACTGGAGAGGGACTACGACAT
2160 G L D S L M G V E V R Q T L E R D Y D I
6721 TGTCATGGCAATGAGGGAAATCCGACAGCTAACCATTAATAAACTACGGGAGCTTTCCAG
2180 V M A M R E I R Q L T I N K L R E L S S
6781 CCAATCAGGAGGGAAAGAGGAGTCCCGAGCTGCTCCTGCAAAGCGATCTAGTGCTCAAGT
2200 Q S G G K E E S R A A P A K R S S A Q V
6841 TCTGCTGGAGTCAGATCTGAGCCGGATGCTGGTGAACCCAGATGACCCCACGGTGGCCCC
2220 L L E S D L S R M L V N P D D P T V A P
6901 ATTGAACGAGGTCCAGAGTGCCGAAAGGCCCTTATTCCTTGTTCACCCCATCGAGGGCTC
2240 L N E V Q S A E R P L F L V H P I E G S
6961 TATTGCAGCCTTCCGCACTCTCACAAGCAAGCTTAGTGTGCCATGCTACGGCCTGCAGTG
2260 I A A F R T L T S K L S V P C Y G L Q C
7021 CACCAAAGCCGCTCCTCTAGACAGTATCCAGAGTCTTGCCGCTTATTACGTGGAGTGCGT
2280 T K A A P L D S I Q S L A A Y Y V E C V
7081 GAGGCAGGTGCAGCCAGAGGGCCCGTATCGTATCGCTGGTTACTCGTTCGGTGCATGTGT
2300 R Q V Q P E G P Y R I A G Y S F G A C V
7141 GGCTTTTGAGATGTGCTCACAGCTGCAGGCAGCCAAATGCCCAGTGGAATACCTCTTCCT
2320 A F E M C S Q L Q A A K C P V E Y L F L
7201 GTTTGACGGCTCGCACTCTTACGTGGCAGCTTATACACAGAGTTACAGAGCAAAACTGAC
2340 F D G S H S Y V A A Y T Q S Y R A K L T
7261 CCCTGGAAAAGAGTCCGAGGCTGAGACTGAAGCTCTGTGTGCTTTTATCCAGCAGTTCAC
2360 P G K E S E A E T E A L C A F I Q Q F T
7321 TGGGATTGAGTACAACAAGCTGTTGGAGACATTGTTACCTCTGTCAGACCTGGAGGCTCG
2380 G I E Y N K L L E T L L P L S D L E A R
7381 TGTGAACAAGGCTGTTAATCTGATCACACCCAGCCATAAGAATGTCAGCCAAGATATGCT
2400 V N K A V N L I T P S H K N V S Q D M L
7441 CCATTTTGCTGCTTCTACATTTTACCACAAACTGAAGGCTGCAGACCGCTATGTTCCAAC
2420 H F A A S T F Y H K L K A A D R Y V P T
7501 TTCTAAGTATCACGGCAATGTCACCCTGCTGAGAGCCAAGGCCAGCAGTGAGTACGGTGA
2440 S K Y H G N V T L L R A K A S S E Y G D
7561 TGGTTTAGGATCTGACTACAAACTGCACGAGGTGTGTGATGGGAAGGTGTCAGTTCATGT
2460 G L G S D Y K L H E V C D G K V S V H V
7621 GATCGAGGGAGACCATCGGACCTTCCTAGAGGGAGAGGGTGTGGAGTCCATCTCAGGCAT
2480 I E G D H R T F L E G E G V E S I S G I
7681 CATCCACAGCTCACTGGCTGAACCCAGAGTCAGCACCAGAGAGGGCTGAACCTTTCTGGC
2500 I H S S L A E P R V S T R E G *
7741 AAACATCTTTTACTTCTTTTTCACCCTTTCTCTGTTGTTTCACCTGATAAACCAAAACTG
7801 TGTCACGGTGTATTAAAGCATGCACGTTTATGATCTCTTCGTACCTCTCACCCACATGAC
7861 AGCCTTTTCCTTCTCCTCTTTCTAATTCTCTTGATATCTTAGTTGCTTATCACCCTTTCC

7921 TTCTCACTTAAAAATTTATATTCCACGCTGAGAACAAAAGCGCATGTCTGTTTACTTGCT
7981 TTGTTTAAGATTCAATCAGCTGTTCATTGTCTTCTATAAATCGTCATACGTTACTGTGAA
8041 AACTAAAATATCACTAATTTCTAGCATGGGAGCATAGTTAAGTGCCAATATGTAGTGATC
8101 ACTGCATAACCTGCATAAGTCTGCTAAAGGTTCTGTCAAAACTGCATTCATGTTCATATG
8161 CATTTCTATATCTAACCTAATGATGCGGACTGCAAGTCAAGGCCTCGCTGTACTCAAATC
8221 CACATATTAGACATCGCGAATCTAGTTGCACACATAGCTGAGATCGTTGTTTACCAATCG
8281 TGAGTGGTTACTGATGCTTTAGAAGTACTAGTTAAAATCGCCTATGTTTACTGTAGCATT
8341 GTTTGATATTTGCTGTTTCTCTTGTTATTGCTGAACATCTAAAATTATGCTGCAATAATT
8401 GTGTATTTAAAAGGGAAGATATGTATGAAAAGAACATTCCTGGATGAGAGTAACCATTGA
8461 GAGATCTGGTTAATTGATTTCTTATTAATGTTTACGTTGAAGTTAGAAGTTAATAATGGA
8521 ATATTTGTTACTTAAAAAATAGATATTGTCTCACTGTGGCACAAGCTAAAAAAAAAATTC
8581 AAATATGACACTATTTTGAAATGCTTGGCAGTCCAGAGTGAATTATCTCGGCTTGTACTG
8641 AAATGATGCTTCACTGCTTAATTGTAGACCAATAACTGGAGTATTAATATTGTATTGACG
8701 TGTCTAATCCTCTTTTTGAGGACTTGTTCCAGGTGTCTCTCAGATGTATATGTTCTCCCA
8761 AAACGATCTGTAATTAATTGACTGCTTTAATACTTAAGGTGTTAGCTGTAGTAGATTTTT
8821 GTGGCCAGGTCCACATTAAACACAAACTCAGTTCATAGTGCAAATCATATTTTAGTCTAA
8881 ATCTGGTCAGAATCAAGGGTTATTTGGTGTAGGAGTGAAACTGGGGATGAATGACTTTAT
8941 CTTTCTGTCACAGCAGAGAAAGGTCTCCTGTTAGAAAGCAAATCACTACACATTTCTGAG
9001 ACTAAGCATTCCAAGTCGGTGACGAATTGTGATTTCTTTCTATGTTTAGAGCAGAGCTGG
9061 GAGAGCTATAGTGTTTGTTTTTCTATTATTGAAATATTTTTTTCTTTTGCTGTAATTTTT
9121 CCAGTATTTATTGTACTAGTCCAAATAAAAAGCTACTGTATTACTAACATAACAC

Figure S1. Nucleotide and deduced amino acid sequence of *Cyprinus carpio var. Jian* FASN gene cDNA. The translation start codon (ATG) and termination codon (TAG) are underlined in the figure. The DM domain is marked with shadow
